# Supplementary figures and images for: Prevalence of TB symptoms, diagnosis and treatment among people living with HIV (PLHIV) not on ART presenting at outpatient clinics in South Africa and Kenya: baseline results from a clinical trial
Source: BMJ Open. 2020 Sep 6;10(9):e035794. doi: 10.1136/bmjopen-2019-035794 (PMC7476481; doi:10.1136/bmjopen-2019-035794)

**Supplementary Fig 1. SLATE I algorithm to support same-day HIV treatment initiation [7]**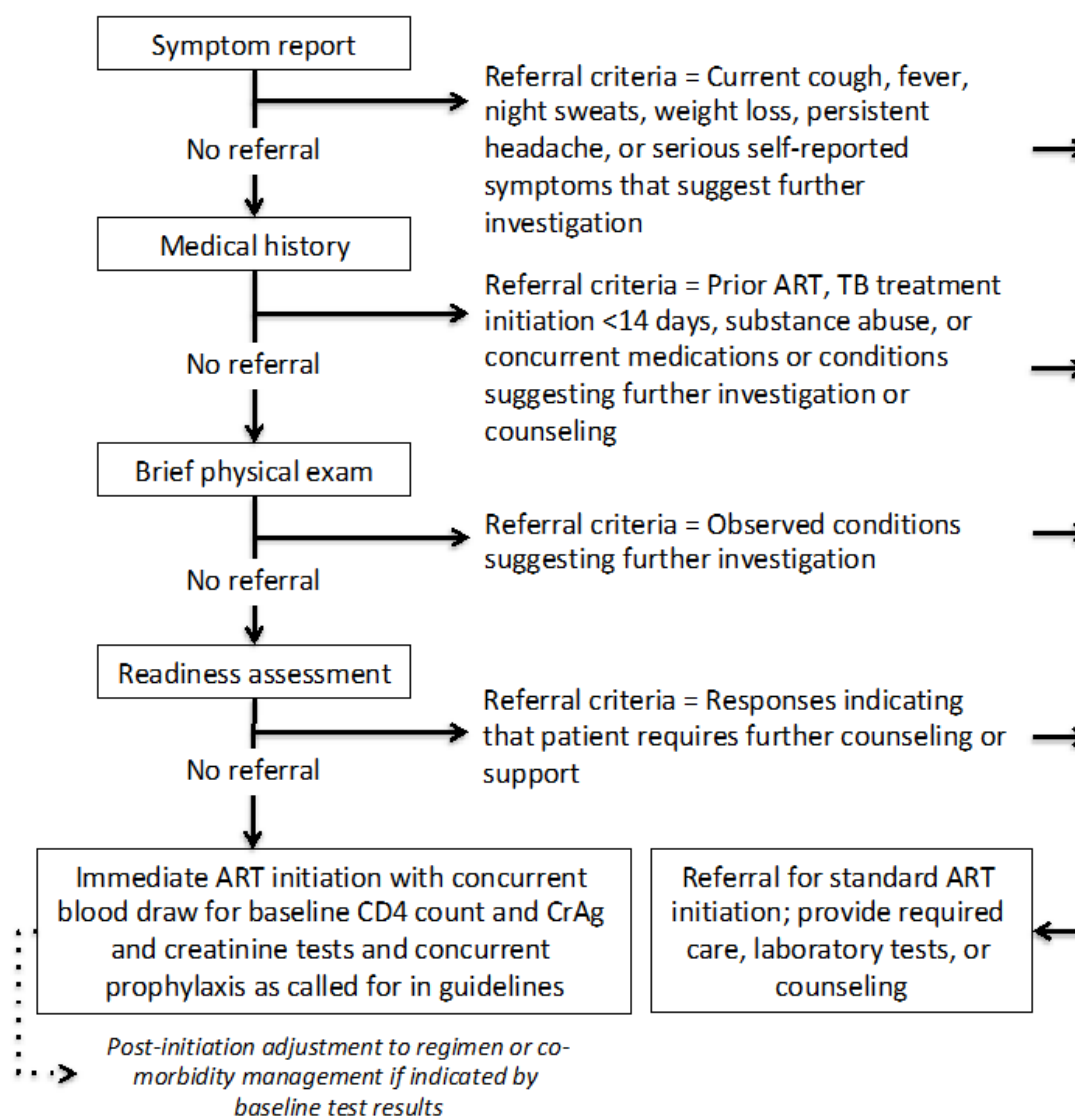

Supplement: Supplementary data [file bmjopen-2019-035794supp001.pdf]

Supplementary Fig 2. SLATE II algorithm to support same-day HIV treatment initiation [8]

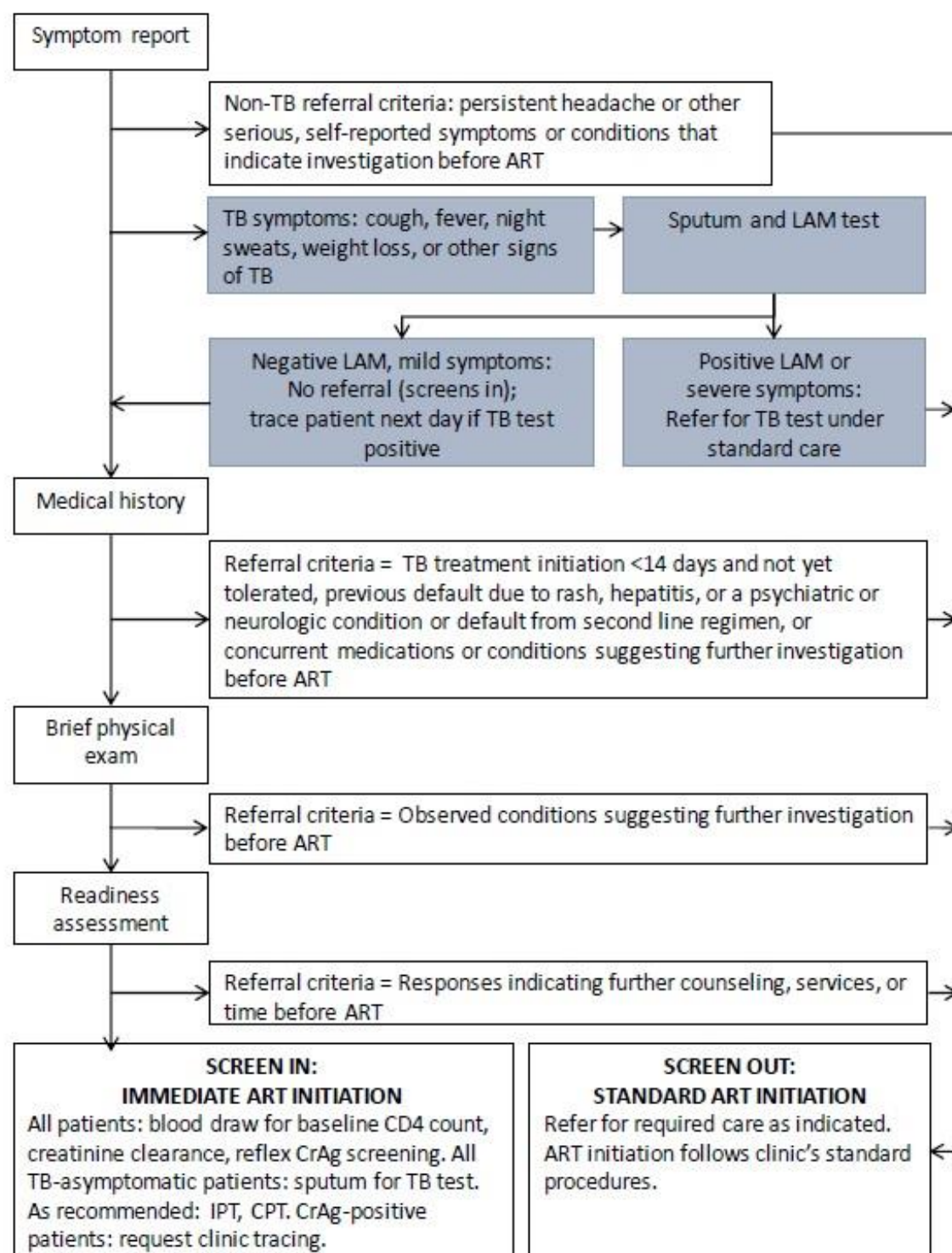

Supplement: Supplementary data [file bmjopen-2019-035794supp002.pdf]
