# Supplementary material for: Prevalence of TB symptoms, diagnosis and treatment among people living with HIV (PLHIV) not on ART presenting at outpatient clinics in South Africa and Kenya: baseline results from a clinical trial
Source: BMJ Open. 2020 Sep 6;10(9):e035794. doi: 10.1136/bmjopen-2019-035794 (PMC7476481; doi:10.1136/bmjopen-2019-035794)
Supplement: Supplementary data [file bmjopen-2019-035794supp003.pdf]

Supplementary Table 1. Characteristics of patients at enrollment in Kenya and South Africa SLATE I and SLATE II trials

**Variables†**	**Categories**	**Intervention arm Kenya (SLATE I) (N = 240)**	**Intervention arm South Africa (SLATE I) (N = 298)**	**Intervention arm South Africa (SLATE II) (N = 296)**
		**n (%)**	**n (%)**	**n (%)**
Period of enrollment		July 13, 2017- April 27, 2018	March 6, 2017- July 28, 2017	March 14- Sept 18, 2018
Sex	Female	142 (59)	189 (63)	189 (64)
Age	Median (IQR)	36 (29, 44)	34 (29, 41)	35 (29, 41)
CD4 count at enrollment (cells/mm^3)	Median (IQR)	272 (124, 522)	275 (132, 459)	294 (135, 464)
Location of current residence	Town (urban)	35 (15)	18 (6)	31 (10)
	Peri-urban	66 (28)	299 (89)	265 (90)
	Rural home or village	139 (58	14 (5)	0 (0)
Current house is primary residence	Yes	154 (64)	141 (47)	128 (43)
Number other persons in house	Median (IQR)	3 (2, 5)	2 (1, 3)	1 (1, 3)
Usual activity when well	Formal employment	18 (8)	82 (28)	69 (23)
	Informal sector work	161 (67)	80 (27)	70 (24)
	Unemployed, looking for work	12 (5)	107 (36)	138 (47)
	Other	9 (4)	29 (10)	12 (4)
Transport mode to clinic today (multiple choices possible)	Minibus or taxi	118 (49)	122 (41)	110 (37)
	Walking	46 (19)	156 (53)	161 (54)
	Private car	11 (5)	19 (19)	25 (8)
	Motorbike	137 (57)	0 (0)	0 (0)

Brennan A, *et al. BMJ Open* 2020; 10:e035794. doi: 10.1136/bmjopen-2019-035794
