# Supplementary material for: Prevalence of TB symptoms, diagnosis and treatment among people living with HIV (PLHIV) not on ART presenting at outpatient clinics in South Africa and Kenya: baseline results from a clinical trial
Source: BMJ Open. 2020 Sep 6;10(9):e035794. doi: 10.1136/bmjopen-2019-035794 (PMC7476481; doi:10.1136/bmjopen-2019-035794)
Supplement: Supplementary data [file bmjopen-2019-035794supp004.pdf]

Supplementary Table 2. Median CD4 count (cells/mm^3) for patients with and without TB symptoms and those with TB diagnosis in SLATE I and SLATE II trials

Variable	Median CD4 Count Intervention arm Kenya (SLATE I)		Median CD4 Count Intervention arm South Africa (SLATE I)		Median CD4 Count Intervention arm South Africa (SLATE II)	
	n (%)	Median (IQR)	n (%)	Median (IQR)	n (%)	Median (IQR)
**No TB symptoms**	148 (62)	357 (191-632)	191 (63)	307 (141-486)	156 (53)	405 (210-547)
**TB symptom positive**	90 (38)	152 (64-329)	105 (37)	245 (125-425)	140 (47)	175 (89-365)
**TB symptom presence**						
Cough (current)	75 (83)	144 (58-319)	71 (68)	274 (132-433)	76 (54)	157 (68-416)
Fever	53 (59)	136 (49-316)	45 (43)	230 (78-368)	20 (14)	110 (73-203)
Night sweats	56 (62)	215 (56-388)	44 (42)	229 (176-368)	29 (21)	135 (101-367)
Weight loss	72 (80)	146 (53-319)	76 (72)	251 (133-433)	96 (69)	157 (68-337)
**TB symptoms**						
1 symptom	13 (14)	207 (147-317)	36 (34)	188 (72-295)	89 (64)	250 (98-369)
2 symptoms	18 (20)	95 (64-232)	27 (26)	373 (227-520)	32 (23)	137 (76-342)
3 symptoms	29 (32)	232 (72-406)	22 (21)	216 (76-368)	8 (6)	165 (63-350)
4 symptoms	30 (33)	116 (24-277)	20 (19)	267 (180-423)	11 (8)	106 (68-157)
**Symptom prevalence in those diagnosed with TB**	**n=12**		**n=9**		**n=7**	
Cough (current)	12 (100)	124 (12-150)	9 (100)	251 (47-288)	6 (86)	92 (60-107)
Fever	9 (75)	114 (12-150)	7 (78)	201 (18-339)	4 (57)	92 (69-107)
Night sweats	12 (100)	142 (55-224)	7 (78)	201 (18-288)	4 (57)	69 (40-92)
Weight loss	10 (83)	124 (12-150)	9 (100)	251 (47-288)	5 (71)	78 (60-106)

Brennan A, *et al. BMJ Open* 2020; 10:e035794. doi: 10.1136/bmjopen-2019-035794
