# Supplementary material for: Prevalence of TB symptoms, diagnosis and treatment among people living with HIV (PLHIV) not on ART presenting at outpatient clinics in South Africa and Kenya: baseline results from a clinical trial
Source: BMJ Open. 2020 Sep 6;10(9):e035794. doi: 10.1136/bmjopen-2019-035794 (PMC7476481; doi:10.1136/bmjopen-2019-035794)
Supplement: Author's manuscript [file bmjopen-2019-035794.draft_revisions.pdf]

BMJ Open is committed to open peer review. As part of this commitment we make the peer review history of every article we publish publicly available.

When an article is published we post the peer reviewers' comments and the authors' responses online. We also post the versions of the paper that were used during peer review. These are the versions that the peer review comments apply to.

The versions of the paper that follow are the versions that were submitted during the peer review process. They are not the versions of record or the final published versions. They should not be cited or distributed as the published version of this manuscript.

BMJ Open is an open access journal and the full, final, typeset and author-corrected version of record of the manuscript is available on our site with no access controls, subscription charges or pay-per-view fees (http://bmjopen.bmj.com).

If you have any questions on BMJ Open's open peer review process please email info.bmjopen@bmj.com

BMJ Open

Prevalence of TB symptoms, diagnosis and treatment among HIV-positive adults in the not yet on ART presenting at outpatient clinics in South Africa and Kenya

Journal:	*BMJ Open*
Manuscript ID	bmjopen-2019-035794
Article Type:	Original research
Date Submitted by the Author:	19-Nov-2019

Complete List of Authors:	Brennan, Alana; Boston University, Departments of Global Health & Epidemiology; Health Economics and Epidemiology Research Office, Department of Internal Medicine, School of Clinical Medicine, Faculty of Health Sciences, University of the Witwatersrand<br>Maskew, Mhairi; Health Economics and Epidemiology Research Office, Department of Internal Medicine, School of Clinical Medicine, Faculty of Health Sciences, University of the Witwatersrand<br>Larson, Bruce; Boston University, Department of Global Health<br>Tsikhutsu, Isaac; Kenya Medical Research Institute; Henry M. Jackson Foundation Medical Research International<br>Bii, Margaret; Kenya Medical Research Institute; Henry M. Jackson Foundation Medical Research International<br>Vezi, Lungi; Health Economics and Epidemiology Research Office, Department of Internal Medicine, School of Clinical Medicine, Faculty of Health Sciences, University of the Witwatersrand<br>Fox, Matt; Boston University, Departments of Global Health & Epidemiology; Health Economics and Epidemiology Research Office, Department of Internal Medicine, School of Clinical Medicine, Faculty of Health Sciences, University of the Witwatersrand<br>Venter, WD Francois; Ezintsha, Wits Reproductive Health and HIV Institute, Department of Internal Medicine, School of Clinical Medicine, Faculty of Health Sciences, University of the Witwatersrand<br>Ehrenkranz, Peter; Bill and Melinda Gates Foundation<br>Rosen, Sydney; Boston University, Department of Global Health; Health Economics and Epidemiology Research Office, Department of Internal Medicine, School of Clinical Medicine, Faculty of Health Sciences, University of the Witwatersrand

Keywords:	Tuberculosis < INFECTIOUS DISEASES, HIV & AIDS < INFECTIOUS DISEASES, Clinical trials < THERAPEUTICS

SCHOLARONE™
Manuscripts

I, the Submitting Author has the right to grant and does grant on behalf of all authors of the Work (as defined in the below author licence), an exclusive licence and/or a non-exclusive licence for contributions from authors who are: i) UK Crown employees; ii) where BMJ has agreed a CC-BY licence shall apply, and/or iii) in accordance with the terms applicable for US Federal Government officers or employees acting as part of their official duties; on a worldwide, perpetual, irrevocable, royalty-free basis to BMJ Publishing Group Ltd ("BMJ") its licensees and where the relevant Journal is co-owned by BMJ to the co-owners of the Journal, to publish the Work in this journal and any other BMJ products and to exploit all rights, as set out in our licence.

The Submitting Author accepts and understands that any supply made under these terms is made by BMJ to the Submitting Author unless you are acting as an employee on behalf of your employer or a postgraduate student of an affiliated institution which is paying any applicable article publishing charge ("APC") for Open Access articles. Where the Submitting Author wishes to make the Work available on an Open Access basis (and intends to pay the relevant APC), the terms of reuse of such Open Access shall be governed by a Creative Commons licence – details of these licences and which Creative Commons licence will apply to this Work are set out in our licence referred to above.

Other than as permitted in any relevant BMJ Author's Self Archiving Policies, I confirm this Work has not been accepted for publication elsewhere, is not being considered for publication elsewhere and does not duplicate material already published. I confirm all authors consent to publication of this Work and authorise the granting of this licence.

For peer review only

Prevalence of TB symptoms, diagnosis and treatment among HIV-positive adults in the not yet on ART presenting at outpatient clinics in South Africa and Kenya

Short title: TB symptoms at ART initiation

Alana T Brennan[1,2,3*], Mhairi Maskew[3*], Bruce A Larson[1§*], Isaac Tsikhutsu[4,5], Margaret Bii[4,5], Lungisile Vezi[3], Matthew P Fox[1,2,3], Willem DF Venter[6], Peter Ehrenkranz[7], Sydney Rosen[1,3*]

1. Department of Global Health, Boston University School of Public Health, Boston, Massachusetts, United States of America
2. Department of Epidemiology, Boston University School of Public Health, Boston, Massachusetts, United States of America
3. Health Economics and Epidemiology Research Office, Department of Internal Medicine, School of Clinical Medicine, Faculty of Health Sciences, University of the Witwatersrand, Johannesburg, South Africa
4. Kenya Medical Research Institute, Kericho, Kenya
5. Henry M. Jackson Foundation Medical Research International, Inc., Nairobi, Kenya
6. Ezintsha, Wits Reproductive Health and HIV Institute, Department of Internal Medicine, School of Clinical Medicine, Faculty of Health Sciences, University of the Witwatersrand, Johannesburg, South Africa
7. Bill & Melinda Gates Foundation, Seattle, Washington, United States of America

*Corresponding author: Alana Brennan, abrennan@bu.edu

Abstract word count: 297
Manuscript word count: 4557

For peer review only

Objective: Using baseline screening data and routine clinic records for intervention arm patients in the Simplified Algorithm for Treatment Eligibility (SLATE) I and II trials, we describe the prevalence of TB symptoms, diagnosis and treatment among HIV-positive adults not on ART presenting at outpatient clinics in sub-Saharan Africa. We also assessed the performance of the World Health Organization (WHO) four-symptom TB screening tool.

Setting: Outpatient HIV clinics in South Africa and Kenya.

Participants: Patients enrolled were non-pregnant, HIV-infected adults $\geq$18 years of age, not currently on ART and willing to provide written informed consent. A total 594 patients in South Africa and 240 in Kenya were eligible.

Results: The prevalence of any TB symptoms was 38% in Kenya, and 35% (SLATE I) and 47% (SLATE II) in South Africa. During SLATE I, 70% of patients in Kenya and 57% in South Africa who had $\geq$1 TB symptom were tested for TB. In SLATE II, 79% of patients with $\geq$1 TB symptom were tested. Of those, 19% tested positive for TB in Kenya. In South Africa, 15% (SLATE I) and 5% (SLATE II) were positive for TB. Of the 28 TB-positive patients in both trials, 20 initiated TB treatment. The lowest median CD4 counts were among those with active TB (Kenya 124 cells/mm^3 (12-150); South Africa 193 cells/mm^3 (56-223)). When comparing the WHO four-symptom screening tool to the Xpert® test, we found increasing the number of symptoms required for a positive screen to $\geq$3 or 4 decreased sensitivity but increased our positive predictive value to >30%.

Conclusions: 80% of patients with $\geq$1 TB symptom were delayed ART initiation for further investigation of TB. Reconsideration of the "any symptom" rule may be appropriate, with ART initiation among patients with fewer/milder TB symptoms commencing while TB test results are pending.

Keywords: Tuberculosis, HIV, Antiretroviral Therapy, Randomized trial, South Africa, Kenya

For peer review only

For peer review only - http://bmjopen.bmj.com/site/about/guidelines.xhtml

Strengths and limitations of this study:

- The World Health Organizations (WHO) current guidelines recommend delaying life-saving antiretroviral therapy (ART) among HIV-positive patients presenting for care with one or more symptoms of tuberculosis (TB)—cough, weight loss, fever, night sweats.
- Over 80% of HIV positive patients with WHO-defined TB symptoms in our study did not have TB, but experienced an unnecessary delay in initiation of ART. Reconsideration of WHO's guidance to even "briefly" delay ART initiation based on presence of "any TB symptom" may be appropriate, with ART initiation among patients with fewer or milder symptoms commencing while TB test results are still pending.
- Study sites were all typical primary healthcare clinics in South Africa and typical hospital-based HIV clinics in Kenya, they were geographically clustered in each country, making generalizability to the rest of the country uncertain.
- The intervention arm of the study was implemented by trained study staff who achieved near-perfect fidelity to intervention procedures; we might not expect such consistent implementation in routine care settings.
- We relied heavily on routinely-collected data pertaining to TB test conduct and results, and it is likely that some TB tests were ordered but not analyzed or analyzed but not recorded. Similarly, data on events after the study enrollment visit, such as post-initiation TB diagnoses or disease, were likely incomplete, and some events may not have been reported to healthcare facilities at all.

For peer review only - http://bmjopen.bmj.com/site/about/guidelines.xhtml

INTRODUCTION

In 2017 the World Health Organization (WHO) began recommending rapid antiretroviral therapy (ART) initiation, including same-day ART initiation (SDI), after the results of several studies indicated that it could reduce loss to follow-up in the pre-ART period[1-3]. The possibility of co-infection with tuberculosis (TB), however, remains a major reason for delaying ART among those with with the TB symptoms on the WHO four-symptom TB screen (cough, weight loss, fever, night sweats). This symptom screen has been shown to have good sensitivity (89%) but poor specificity (28%) in ART naïve HIV-positive patients[4]. According to the WHO and national guidelines in both South Africa[5] and Kenya[6], patients who report ≥1 TB symptoms require further investigation for active TB disease before ART initiation, which entails a laboratory test such as Xpert® MTB/RIF. Following the TB test, patients with negative results resume regular procedures for ART initiation, while those found to have TB are started on TB therapy, with ART initiation delayed until patients are regarded as stable on TB treatment. As a second clinic visit is typically required to receive TB test results, SDI may be impossible for patients presenting with TB symptoms.

The Simplified Algorithm for Treatment Eligibility (SLATE) I study in South Africa and Kenya[7] and the SLATE II study in South Africa[8] evaluated a clinical algorithm to assess eligibility for rapid ART initiation in patients presenting for HIV care but not currently on ART. The algorithms distinguished between patients eligible for SDI of ART and patients requiring referral to clinic staff for additional standard of care evaluation and TB treatment before ART initiation. One or more symptoms of TB—cough, fever, weight loss or night sweats—of any duration or intensity were among the criteria for referral in SLATE I. SLATE II revised the SLATE I algorithm to allow patients with mild TB symptoms and a negative lipoarabinomannan assay(LAM) test[9] to initiate on the same day, based on clinician judgement.

The purpose of this analysis is to describe the prevalence of TB symptoms among patients presenting for HIV care but not currently on ART and to estimate rates of TB testing, diagnosis, and treatment using baseline screening data and routine clinic records for intervention arm patients in the SLATE I and

For peer review only - http://bmjopen.bmj.com/site/about/guidelines.xhtml

II clinical trials in South Africa and Kenya. We also assessed the performance of the WHO four-symptom TB screening tool compared to Xpert® MTB/RIF sputum testing. The implications of current TB screening, diagnosis, and treatment for implementation of SDI are discussed.

METHODS

SLATE trials

The SLATE I and SLATE II trials in South Africa and Kenya were multicenter trials evaluating two variations of a simplified algorithm to determine eligibility for SDI of ART without relying on laboratory results or multiple clinic visits[7, 8]. Enrollment for SLATE I (NCT02891135) was completed in July 2017 in South Africa and April 2018 in Kenya. Enrollment for SLATE II (NCT03315013), only conducted in South Africa, was completed in September 2018.

Study population

Patient inclusion and exclusion criteria for study eligibility have been described previously [7,8]. Briefly, those enrolled were non-pregnant, HIV-infected adults $\geq$18 years of age, not currently on ART and willing to provide written informed consent, who were randomized 1:1 to the intervention and standard of care arms. This analysis is limited to patients randomized to the intervention arm, for whom we have TB symptom data. Symptom screening of patients in the standard of care arm was poorly documented by clinic staff. Intervention arm patients were assessed for eligibility for SDI by a study nurse (South Africa) or clinical officer (Kenya) using the SLATE I or II algorithm, which each consisted of four screens: (1) current symptoms, (2) recent medical history, (3) physical conditions, and (4) treatment readiness (see Supplementary Figure 1 and Supplementary Figure 2). The baseline characteristics of these patients have previously been described[10].

Approval of the SLATE trials was provided by the Human Research Ethics Committee of the University of the Witwatersrand in South Africa (SLATE I 160910; SLATE II 171011), the Kenya Medical Research Institute (SLATE I 3408) and the Walter Reed Army Institute of Research in Kenya (SLATE I 2401) , and the Institutional Review Board of Boston University (SLATE I H-35634; SLATE II H-37010).

For peer review only - http://bmjopen.bmj.com/site/about/guidelines.xhtml

Patients were not involved in the design, recruitment or conduct of the SLATE trials. We conducted a qualitative study at the end of SLATE II to help gain a deeper understanding of patient perceptions of initiating ART per standard of care compared to same-day initiation using the SLATE algorithm. Presentation of primary study results will be conducted by study staff at participating clinics to disseminate research findings to staff and patients prior to funding ending in July 2020.

TB screening and diagnosis differences between SLATE I and SLATE II

The main difference between the algorithm used in SLATE I and the revised version used in SLATE II was the approach to TB screening and diagnosis. Both algorithms asked patients for self-reported TB symptoms (cough, fever, weight loss, or night sweats), based on the WHO four-symptom screen[11]. Procedures differed from that point forward, as described below and illustrated in supplementary figures 1 and 2.

In SLATE I, consistent with national guidelines in both countries, patients reporting any symptoms of TB of any severity or duration were referred to routine clinic care, which should have included a TB test according to guidelines. Each patient was given a referral letter for the clinic indicating the reason for referral, such as the presence of TB symptoms, but no further effort was made by study staff to ensure that the patient remained in care or was tested or treated for TB.

As has previously been reported[12], in SLATE I and illustrated in Figures 1 and 2, a larger proportion of intervention arm patients than expected were ineligible for SDI due to TB symptoms (38% in Kenya and 37% in South Africa). The presence of $\geq$1 more TB symptoms was by far the main reason for ineligibility under the SLATE I algorithm. Very few of these patients were ultimately confirmed to have TB, however, and study patients reported no TB-related adverse events after starting ART. We thus speculated that referring a patient out for mild TB symptoms, without further complications, was too stringent a requirement, and we developed the SLATE II algorithm accordingly.

In SLATE II, if a patient reported $\geq$1 TB symptoms, the newly developed TB module in the algorithm was applied. The TB module included: (1) a more detailed medical history (e.g. inquiring about severity and

For peer review only - http://bmjopen.bmj.com/site/about/guidelines.xhtml

duration of symptoms); (2) a focused physical examination to assess TB symptoms; (3) sputum collection for Xpert® MTB/RIF sputum testing; and (4) a point-of-care, urine-based LAM test[9]. Patients with a positive LAM test, TB symptoms that were severe or of long duration, or any other clinical finding indicating active TB were referred back to routine care under the SLATE II algorithm. As with SLATE I, no effort was made by study staff to ensure that the patient actually underwent a TB test once referred back to routine care. A patient with a negative LAM test and without severe symptoms of TB remained eligible for same day initiation of ART. For SLATE II, unlike SLATE I, the study staff collected a sputum sample from all intervention arm patients who were able to produce one, regardless of TB symptoms. These samples were sent to the National Health Laboratory Service for Xpert® MTB/RIF sputum testing for TB disease.

Data collection

We collected demographic and algorithm data for intervention arm patients via a case report form administered at study enrollment. Laboratory tests results (e.g., CD4 counts and Xpert® MTB/RIF findings) were extracted directly from laboratory electronic records or paper-based registers kept at each site, while follow-up data for the study period were collected from routinely generated clinical record data from patient records in electronic and paper format.

Statistical analysis

Simple descriptive statistics were used to display demographic and clinical characteristics of patients at study enrollment, prevalence of TB symptoms, TB diagnosis and TB treatment.

Having Xpert® MTB/RIF sputum tests on the majority (n=214) of SLATE II intervention arm patients provided a unique opportunity to assess the performance of the WHO four-symptom TB screening tool. We defined 11 possible interpretations of TB symptom screening results and compared them to the gold standard of Xpert® MTB/RIF: 1) any TB symptom; 2) $\geq$2 symptoms; 3) $\geq$3 TB symptoms; 4) all 4 TB symptoms; 5) cough alone; 6) fever alone; 7) weight loss alone; 8) night sweats alone; 9) cough and fever; 10) cough and night sweats and 11) cough and weight loss. We calculated the sensitivity (probability of screening positive (using each definition above) when TB disease is present as defined

For peer review only - http://bmjopen.bmj.com/site/about/guidelines.xhtml

by Xpert® MTB/RIF), specificity (probability of screening negative when TB disease is not present as defined by Xpert® MTB/RIF), positive predictive value (probability of a patient having TB disease when the screen is positive) and negative predictive value (the probability of a patient not having TB disease when the screen is negative).

RESULTS

Demographic and clinical characteristics

Supplementary Table 1 summarizes basic characteristics of intervention arm patients stratified by trial cohort (N=240 for SLATE I Kenya; N=298 for SLATE I South Africa; N=296 for SLATE II). The majority of patients in each group were females in their mid-thirties, with a median baseline CD4 count between 272 and 294 cells/mm^3. We excluded 4 patients in Kenya and 1 in South Africa in the SLATE I trial who were known to be on TB treatment at study enrollment (Table 1). Further details of baseline characteristics of the cohorts have been reported previously[10].

TB symptom prevalence

The prevalence of any TB symptoms was 38% [95% confidence interval:32-44%] in SLATE I in Kenya, 35% [30-41%] in SLATE I in South Africa, and 47% [42-53%] in SLATE II (Table 1). In both studies and both countries, amongst people with any symptom, cough (66% combined) and weight loss (72% combined) were the most common symptoms reported. As presented in Supplementary Table 2, patients with TB symptoms had substantially lower CD4 counts in all three cohorts at study enrollment than did those with no symptoms of TB, indicating more advanced HIV disease among symptomatic patients. We saw little variation in CD4 count when the data were stratified by number of symptoms or symptom type. The lowest median CD4 counts were recorded among those found to have active TB disease (Kenya 124 cells/mm^3 [12-150]; South Africa (SLATE I and II combined) 193 cells/mm^3 [56-223].

TB testing and diagnosis among symptomatic patients

In Kenya (SLATE I), 90 (38% [32-44%]) intervention arm patients had $\geq$ 1 symptom of TB and screened out of the algorithm, with referral back to standard care. Clinic staff chose or were able to test only 63/90 (70% [60-79%]) symptomatic patients for TB. Of those tested, 12/63 (19% [11-30%]) had a

For peer review only - http://bmjopen.bmj.com/site/about/guidelines.xhtml

positive result, corresponding to an estimated TB prevalence in symptomatic patients presenting for care in Kenya of 5% [3-8%] (assuming those not tested were negative for TB disease) (Figure 1). In SLATE I in South Africa, we saw a similar proportion of patients presenting with at least 1 symptom of TB (105, 35% [30-41%]), but only 60 (57% [48-66%]) of these symptomatic patients were tested for TB by the study clinics. Of those tested, 9/60 (15% [8-26%]) had a positive result, corresponding to an estimated TB prevalence in patients presenting for care in South Africa during SLATE I of 3% [2-5%] (Figure 2), with the same assumption regarding the negative status of those not tested.

In SLATE II, all 296 intervention arm patients were asked for a sputum sample per the study protocol[8], regardless of symptoms. We were able to successfully collect and test sputum for 111 (79% [72-85%]) symptomatic patients and 118 (76% [68-82%]) asymptomatic patients, or 72% (n=214/296) overall (Figure 3). Of the 29 symptomatic patients not tested, 25 were unable to produce a sputum sample and 4 refused to test. Among the 106 symptomatic patients in SLATE II with a successful test, 6 (5% [2-11%]) results were positive for TB, producing an estimated TB prevalence in all HIV patients (asymptomatic and symptomatic) presenting for care in South Africa during SLATE II of 2% [1-5%], with the same assumption regarding the negative status of those not tested.

Amongst the total of 28 TB-positive patients in both studies, 92% of patients in Kenya and 81% in South Africa (SLATE I and II combined) had at least three symptoms of the disease, and 67% in Kenya and 63% in South Africa had all four symptoms. Virtually all (96% [84-99%]) those diagnosed presented with a cough. Weight loss was also common (86% [69-95%]), followed by night sweats (82% [65-93%]) and fever (71% [53-86%]) (Table 1).

TB testing and diagnosis in asymptomatic patients

Among the 337 (63% [58-67%]) patients with no TB symptoms in SLATE I, four (3% [1-6%]) patients in Kenya and 33 (17% [12-23%]) patients in South Africa were tested for TB by the study clinics. In Kenya, three of the patients had documented reasons for being tested by the clinic (one was taking cough syrup for the previous 5 days and was recently screened for TB, one had suspected extra-pulmonary TB due to swelling in the jaw, and one patient was asthmatic). The remaining patient had no documented

For peer review only - http://bmjopen.bmj.com/site/about/guidelines.xhtml

reason for a TB test. In South Africa, one of the clinics in our study was attempting to collect sputum from all HIV positive patients prior to ART initiation, regardless of symptoms. None of the 33 asymptomatic patients tested were positive (Figures 1 and 2).

In SLATE II, as per the study protocol[8], all 296 intervention arm patients were asked for a sputum sample. We were able to successfully collect and test sputum for 118 (76% [68-93%]) asymptomatic patients (Figure 3). One positive TB test result was recorded among those who were eligible for SDI under the SLATE II algorithm. This was an asymptomatic patient who had a negative LAM test and CD4 count of 350 cells/mm^3. The patient was successfully traced and commenced TB treatment the following day.

TB LAM test results

In SLATE II, all intervention arm subjects with ≥1 symptoms of TB had a LAM test performed. Only two tests (<1%) were positive. Both patients had sputum samples taken for Xpert® testing. The first patient was a male who came to the clinic for care because he felt unwell. He reported all four TB symptoms and had a CD4 count of 78 cells/mm^3. This patient's result came back positive for TB and he was successfully traced and commenced on TB treatment. The second patient was a female who came for an HIV test and reported cough and weight loss and had a CD4 count of 19 cells/mm^3. This patient tested negative for TB on Xpert®.

TB treatment uptake

In Kenya, eight of the 12 patients (67% [38-88%]) who tested positive for TB went on to initiate treatment for the disease, all within two weeks of diagnosis. Of the remaining four patients, one died within three weeks of study enrollment with no record of starting TB treatment, and the remaining three remained in care but had no record of initiating TB treatment. Of the 16 patients (symptomatic and asymptomatic) who tested positive in SLATE I and II in South Africa, eight initiated TB treatment after diagnosis (four patients within one week and four within seven weeks after study enrolment), four patients remained in care but had no record of starting TB treatment, three patients were

For peer review only - http://bmjopen.bmj.com/site/about/guidelines.xhtml

transferred to another facility before starting TB treatment, and one patient was lost from care before starting treatment.

Unmasking TB disease

One of the major concerns about SDI is immune reconstitution inflammatory syndrome among patients with undiagnosed TB (TB-IRIS). No patients in either study (both standard of care and intervention arms) had indications in their routine clinic records of IRIS reactions during passive study follow up, though this may reflect incomplete record-keeping. Patient clinical records indicated that clinic staff investigated a total of 25 patients for TB more than 30 days after study enrollment. Of these, 24 test results were available (we were unable to locate one test result in SLATE I in South Africa) and two were positive for TB disease (one positive patient was in the standard of care arm in South Africa (SLATE I) and one was in the intervention arm in Kenya (SLATE I) and not eligible for SDI). Among these 24 patients with a follow-up TB test, 4 (17%) (three in SLATE I and one in SLATE II) were eligible for SDI via the SLATE algorithm, none of these patients had a positive TB test.

In the rest of the results section, we will focus solely on SLATE II in South Africa, where the new TB module in the SLATE II algorithm was applied.

TB in symptomatic patients eligible for SDI under SLATE II algorithm

In the SLATE II study, patients with milder TB symptoms and a negative TB LAM test were eligible for SDI under the study at the discretion of the study nurse. Of the intervention arm patients who screened in and were eligible for SDI, 40% [34-46%] (n=101) presented with at least one of the four TB symptoms. Of these, 76% [66-83%] (n=77) reported only one symptom, 20% [13-28%] (n=20) reported two symptoms, and 4% [1-9%] (n=4) reported 3 or more symptoms. The most frequently reported symptoms were weight loss (64%) and cough (45%); night sweats (13%) and fever (6%) were less frequently reported. Of the 101 symptomatic patients, 75% [64-81%] (n=74) successfully completed a TB test (the remainder either could not produce a sputum sample or were unwilling to do so) and of these, only one tested positive for TB. This patient reported only cough and had a negative LAM test during administration of the SLATE II algorithm. After SDI, the patient, whose CD4 count was 116

For peer review only - http://bmjopen.bmj.com/site/about/guidelines.xhtml

cells/mm^3, could not be traced to start TB treatment, despite phone calls and a home visit. The patient never returned to the clinic after study enrolment for either TB or HIV care.

Sensitivity, specificity and predictive values of SLATE II algorithm

Figure 4 displays the results of the performance of the WHO four-symptom screen in SLATE II intervention arm patients. The sensitivity and specificity of any single TB symptom were 86% [47-99%] and 52% [45-59%], respectively. The positive predictive value was 6% [2-12%], while the negative predictive value was 99% [96-99%]. When modifying the definition of a positive TB screen to $\geq$3 symptoms, sensitivity decreased to 71% [33-95%] and specificity increased to 95% [92-98%]. The positive predictive value improved to 33% [13-59%] while negative predictive value remained unchanged (99% [97-99%]). The WHO symptom screening tool appeared to perform the best for sensitivity (86% [47-99%]) and specificity (72% [66-78%]) when using cough only. However, the positive predictive value was the highest in the analysis using $\geq$3 (33% [13-59%]) and 4 (40% [14-71%]) symptoms. In all other analyses of the WHO four-symptom screen, sensitivity falls, and specificity increases as a result, while the positive predictive value ranges from 7% to 31% depending on how the prevalence shifts. The negative predictive value remained unchanged for all analyses at above 98%. This was expected as the prevalence of TB in the population was low (3%) based on Xpert® testing.

DISCUSSION

In three cohorts of HIV-positive patients not yet on ART and presenting for care in Kenya and South Africa since 2017, we estimated an overall prevalence of any TB symptoms of 35% to 50%. Between 60% and 80% of these patients were tested for TB, among whom 19% in Kenya and 6% to 15% in South Africa had a positive TB test, corresponding to an estimated prevalence in patients presenting for care of 5% in Kenya and 2% to 3% in South Africa (assuming those not tested are negative for TB disease). The results we report for TB symptom prevalence (with cough also being the most common symptom reported) and percentage of patients with TB symptoms tested for TB reported in our study are consistent with what has been previously published at a national level in Kenya[13] and in HIV study cohorts in South Africa[14-21]. The consistency of our estimates of TB disease prevalence with

For peer review only - http://bmjopen.bmj.com/site/about/guidelines.xhtml

previously published literature varied. Some studies report estimates that were comparable[13,14], lower[15], or higher[16-21]. National estimates of TB prevalence are still sparse in South Africa.

In our studies, over 80% of the 28 patients who tested positive for TB disease reported having at least three clinical symptoms of the disease, the most common being a cough, followed by weight loss, night sweats, and fever. Other studies have estimated more than 80% of patients diagnosed with TB had more than one symptom of the condition[18,19,23]. A study in South Africa also reported that more than 80% of patients without TB disease had symptoms [22], again consistent with our findings.

Using SLATE II results, the performance of the WHO four-symptom screen tool, when classifying patients with ≥1 symptoms of TB, was comparable regarding sensitivity to what was reported in a recent meta-analysis[4], but we had a higher specificity at 52% (vs. 28%[4]). When we increased the number of symptoms required for a positive screen to ≥3 or 4, we saw a decrease in sensitivity and subsequent increase in specificity and an increase in the positive predictive value from 6% (for ≥1 symptoms of TB) to 33% and 40%, respectively.

When delaying ART in an HIV patient is contingent on a positive TB test, the positive predictive value is what is more clinically relevant. The main reason for the delay of ART initiation in TB suspect patients is to prevent TB-IRIS. The estimated risk of TB-IRIS is quite low, however, at roughly 1% to 6% in sub-Saharan Africa[24]. Additionally, the risk is the highest in patients presenting with CD4 counts <100 cells/mm^3[25,26]. The median CD4 count in all three of our cohorts amongst symptomatic patients was above 100 cells/mm^3, while patients presenting with no symptoms had a median CD4 count above 300 cells/mm^3. The lowest median CD4 counts, and those at highest risk of TB-IRIS, were amongst patients that had three if not all four symptoms of TB disease and had a positive Xpert® test. All of which were delayed ART initiation for further investigation of TB disease. In a healthcare system that can produce TB test results soon after ART initiation and successfully contact (trace) patients with positive results, it may be reasonable to increase the number of TB symptoms required to trigger a delay in ART initiation to 3 or 4. This would increase the probability that a patient who is classified as TB positive (using the WHO four-symptom screen) truly has TB disease and allow patients with fewer or milder TB symptoms

For peer review only - http://bmjopen.bmj.com/site/about/guidelines.xhtml
For peer review only

to start ART while TB test results are pending. It is still unknown whether initiating ART prior to TB being confirmed or treated causes harm (or benefit). In a setting in which TB tests are delayed and/or active tracing of patients with positive results is poor, in contrast, a more conservative approach—delaying ART until a TB test can be completed for patients with even one symptom—may continue to be justified. However, more research is required to accurately weigh the risks of delaying ART (which might prevent a person from being successfully linked into HIV treatment) versus the benefits of waiting for a definitive TB diagnosis to minimize the already low and potentially manageable clinical risk of TB-IRIS.

In both Kenya and South Africa, national TB guidelines during the study stated that HIV patients self-reporting any TB-related symptoms should be tested with Xpert® MTB/RIF and treated if diagnosed with the disease. In SLATE I, we saw gaps in following national guidelines in both countries. Twenty-seven symptomatic, intervention arm patients in Kenya and 45 in South Africa were ineligible for SDI due to TB symptoms and were referred back to the clinic for further testing but were not tested for TB by the clinic staff. We assume that a certain number of patients refused or were unable to provide a sputum sample for testing, but for some, the nurse or clinical officer who saw the patient chose not to do a test. At one study site in South Africa we were told, informally, that staff only requested TB tests if two or more symptoms were present, while at one site in Kenya, a clinical officer would diagnose a respiratory infection before TB and require the patient to go through a course of antibiotics, advising the patient to return for a TB test only if symptoms persisted. Whether failure to follow guidelines precisely reflects reasonable use of clinical judgment, lack of available resources, or irresponsible non-compliance on the part of clinic staff is unclear, but it should be considered in efforts to improve treatment of TB disease and maximize opportunities to offer TB preventative treatment for patients without TB.

The urine TB-LAM test was positive in <1% of our intervention arm patients in the SLATE II trial. As the TB-LAM has been shown to improve TB diagnosis in patients with low CD4 counts[27] this could be due to higher CD4 counts in our cohort. The average CD4 count in SLATE II intervention arm patients was 294 cells/mm^3, it was 175 cells/mm^3 amongst those with TB symptoms and ranged from 60-107

For peer review only - http://bmjopen.bmj.com/site/about/guidelines.xhtml

cells/mm^3 amongst those diagnosed with TB. Also, previous studies have shown that the positive predictive value of urine LAM testing depends upon the prevalence of TB disease in the population, which was low (6%) in our SLATE II cohort[28]. Given the TB-LAM test is part of the care package under current WHO guidelines, it may not offer much benefit for the cost involved.

Our study had several limitations. First, while the study sites were all typical primary healthcare clinics in South Africa and typical hospital-based HIV clinics in Kenya, they were geographically clustered in each country, making generalizability to the rest of the country uncertain. Second, the intervention arm of the study was implemented by trained study staff who achieved near-perfect fidelity to intervention procedures; we might not expect such consistent implementation in routine care settings. Third, we relied heavily on routinely-collected data pertaining to TB test conduct and results, and it is likely that some TB tests were ordered but not analyzed or analyzed but not recorded. Similarly, data on events after the study enrollment visit, such as post-initiation TB diagnoses or disease, were likely incomplete, and some events may not have been reported to healthcare facilities at all. Finally, our sample sizes were small, limiting our ability to stratify by patient characteristics; a larger sample might identify other characteristics associated with a positive TB test. The small sample size could also affect our ability to detect TB-related adverse events in our sample.

Despite these limitations, we conclude that in the SLATE I and SLATE II trials, among 235 patients with WHO-defined TB symptoms who were not eligible for SDI, over 80% did not have TB but experienced an unnecessary delay in ART initiation. No serious, TB-related adverse events were reported after starting ART among symptomatic patients with or without delay in our study. Reconsideration of WHO's guidance to even "briefly" delay ART initiation based on presence of "any TB symptom" may be appropriate, with ART initiation among patients with fewer or milder symptoms commencing while TB test results are still pending.

Acknowledgments

We thank the patients who participated in the study and the staff of the six study clinics for their cooperation; the City of Johannesburg and Ekhureleni Metro in South Africa and the management of

For peer review only - http://bmjopen.bmj.com/site/about/guidelines.xhtml

Kericho County Referral Hospital, Kapsabet County Referral Hospital, and Kombewa County Hospital in Kenya for permission to conduct this study.

Contributors: AB, MM and BL cleaned and analysed the data. All the authors contributed substantially to the interpretation of the results and the writing of the manuscript. All the authors have read and approved the final version of the manuscript.

Competing interests: None declared.

Funding: Research was funded by the Bill and Melinda Gates Foundation (Grant No. OPP1136158)

Data sharing statement: Data generated by the study will be made publicly available in the Dryad repository (http://www.datadryad.org/) after the protocol has been closed (anticipated closure July 2020). Until then, data will remain under the supervision of the Boston University Medical Campus IRB, the University of the Witwatersrand Human Research Ethics Committee (HREC), and the KEMRI Scientific and Ethics Research Unit. Requests can be sent to the BUMC IRB at medirb@bu.edu. Data extracted from routine medical records are owned by the study sites, the South African National Department of Health, and the Kenyan Ministry of Health and cannot be made publicly available by the authors.

For peer review only

For peer review only - http://bmjopen.bmj.com/site/about/guidelines.xhtml

References

1. Guidelines for Managing Advanced HIV Disease and Rapid Initiation of Antiretroviral Therapy. Geneva: World Health Organization; 2017.
2. Amanyire G, Semitala FC, Namusobya J, Katuramu R, Kampiire L, Wallenta J et al. Effects of a multicomponent intervention to streamline initiation of antiretroviral therapy in Africa: a stepped-wedge cluster-randomised trial. Lancet HIV. 2016;3:e539–48.
3. Rosen S, Maskew M, Fox MP, Nyoni C, Mongwenyana C, Malete G et al. Initiating antiretroviral therapy for HIV at a patient's first clinic visit: the RapIT Randomized Controlled Trial. PLoS Med. 2016;13:e1002015.
4. Hamada Y, Lujan J, Schenkel K, Ford N, Getahun H. Sensitivity and specificity of WHO's recommended four-symptom screening rule for tuberculosis in people living with HIV: a systematic review and meta-analysis. Lancet HIV. 2018 Sep;5(9):e515-e523.
5. National Department of Health. Same-day antiretroviral therapy (ART) initiation for HIV positive patients. Pretoria: National Department of Health; 2017.
6. Ministry of Health and National AIDS and STI Control Program (NASCOP). Guidelines on the Use of Antiretroviral Drugs for Treating and Preventing HIV Infection in Kenya 2018 Edition. Nairobi, Kenya; 2018.
7. Rosen S, Fox MP, Larson BA, Brennan AT, Maskew M, Tsikhutsu I, Bii M, Ehrenkranz PD, Venter WF. Simplified clinical algorithm for identifying patients eligible for immediate initiation of antiretroviral therapy for HIV (SLATE): protocol for a randomised evaluation. BMJ Open. 2017 May 28;7(5):e016340.
8. Rosen S, Maskew M, Brennan AT, Fox MP, Vezi L, Ehrenkranz PD, Venter WDF. Improved simplified clinical algorithm for identifying patients eligible for immediate initiation of antiretroviral therapy for HIV (SLATE II): protocol for a randomized evaluation. Trials. 2018 Oct 11;19(1):548.
9. World Health Organization. The use of lateral flow urine lipoarabinomannan assay (LF-LAM) for the diagnosis and screening of active tuberculosis in people living with HIV TB. Geneva: World Health Organization, 2015.
10. Brennan A, Maskew M, Larson B, Tsikhutsu I, Bii M, Vezi L, Fox M, Venter F, Ehrenkranz P, Rosen S. Who is seeking antiretroviral treatment for HIV now? Characteristics of patients presenting in Kenya and South Africa in 2017-2018. J Int AIDS Soc; in press.
11. World Health Organization. Systematic screening for active tuberculosis Principles and recommendations. Geneva: World Health Organization, 2013.
12. Maskew M, Larson B, Brennan AT, Fox MP, Vezi L, Tsikhutsu I, Bii M, Venter WDF, Rosen S. Same-day ART initiation without laboratory tests is safe and effective: primary outcomes of the SLATE trial in South Africa and Kenya. PLoS Medicine. 2019.
13. Kenya Tuberculosis Prevalence Survey. Republic of Kenya. Minisrty of Health. 2016. Available at: https://www.chskenya.org/wp-content/uploads/2018/04/Final-TB-Prevalence-Survey-Report.pdf.
14. Rangaka MX, Wilkinson RJ, Glynn JR, Boulle A, van Cutsem G, Goliath R, et al. Effect of antiretroviral therapy on the diagnostic accuracy of symptom screening for intensified tuberculosis case finding in a south African HIV clinic. Clin Infect Dis. 2012;55(12):1698–706.

For peer review only - http://bmjopen.bmj.com/site/about/guidelines.xhtml

15. Hanrahan CF, Nonyane BAS, Mmolawa L, West NS, Siwelana T, Lebina L, Martinson N, Dowdy DW. Contact tracing versus facility-based screening for active TB case finding in rural South Africa: A pragmatic cluster-randomized trial (Kharitode TB). PLoS Med. 2019 Apr 30;16(4):e1002796.
16. Bajema KL, Bassett IV, Coleman SM, Ross D, Freedberg KA, Wald A, Drain PK. Subclinical tuberculosis among adults with HIV clinical features and outcomes in a South African cohort. BMC Infect Dis. 2019 Jan 5;19(1):14. doi: 10.1186/s12879-018-3614-7. PubMed PMID: 30611192; PubMed Central PMCID: PMC6321698.
17. Bassett IV, Wang B, Chetty S, Giddy J, Losina E, Mazibuko M, et al. Intensive tuberculosis screening for HIV-infected patients starting antiretroviral therapy in Durban. South Africa Clin Infect Dis. 2010;51(7):823–9.
18. Kerkhoff AD, Wood R, Lowe DM, Vogt M, Lawn SD. Blood neutrophil counts in HIV-infected patients with pulmonary tuberculosis: association with sputum mycobacterial load. PLoS One. 2013;8(7):e67956.
19. Ahmad Khan F, Verkuijl S, Parrish A, Chikwava F, Ntumy R, El-Sadr W, et al. Performance of symptom-based tuberculosis screening among people living with HIV: not as great as hoped. AIDS. 2014;28(10):1463–72.
20. Lawn SD, Brooks SV, Kranzer K, Nicol MP, Whitelaw A, Vogt M, et al. Screening for HIV-associated tuberculosis and rifampicin resistance before antiretroviral therapy using the Xpert MTB/RIF assay: a prospective study. PLoS Med. 2011;8(7):e1001067.
21. Kranzer K, Houben RM, Glynn JR, Bekker LG, Wood R, Lawn SD. Yield of HIV-associated tuberculosis during intensified case finding in resource-limited settings: a systematic review and meta-analysis. Lancet Infect Dis. 2010 Feb;10(2):93-102.
22. Human Sciences Research Council (HSRC). National Tuberculosis Prevalence Survey underway. February 2018. Available at: http://www.hsrc.ac.za/en/media-briefs/saph/national-tb-prevalance-survey-underway
23. Swindells S, Komarow L, Tripathy S, Cain KP, MacGregor RR, Achkar JM, et al. Screening for pulmonary tuberculosis in HIV-infected individuals: AIDS Clinical Trials Group protocol A5253. Int J Tuberc Lung Dis. 2013;17(4):532–9.
24. Walker NF, Stek C, Wasserman S, Wilkinson RJ, Meintjes G. The tuberculosis-associated immune reconstitution inflammatory syndrome: recent advances in clinical and pathogenesis research. Curr Opin HIV AIDS. 2018 Nov;13(6):512-521.
25. Manabe YC, Campbell JD, Sydnor E, Moore RD. Immune reconstitution inflammatory syndrome: risk factors and treatment implications. J Acquir Immune Defic Syndr. 2007 Dec 1;46(4):456-62.
26. Cain KP, McCarthy KD, Heilig CM, Monkongdee P, Tasaneeyapan T, Kanara N, et al. An algorithm for tuberculosis screening and diagnosis in people with HIV. N Engl J Med. 2010 Feb 25;362(8):707-16.
27. Huerga H et al. Diagnostic value of the urine lipoarabinomannan assay in HIV-positive, ambulatory patients with CD4 below 200 cells/µl in 2 low-resource settings: a prospective observational study. PLoS Medicine 16(4): e1002792, 2019.

For peer review only - http://bmjopen.bmj.com/site/about/guidelines.xhtml

28. Drain PK, Gounder L, Sahid F, Moosa MY. Rapid Urine LAM Testing Improves Diagnosis of Expectorated Smear-Negative Pulmonary Tuberculosis in an HIV-endemic Region. Sci Rep. 2016 Feb 11;6:19992.

For peer review only

For peer review only - http://bmjopen.bmj.com/site/about/guidelines.xhtml

Table 1. Self-reported TB symptoms, TB diagnosis, and TB treatment uptake among patients who screened out due to TB symptoms in the intervention arms of the SLATE I and SLATE II trials

Variable (% responding yes)	Kenya (SLATE I) (N = 240)	South Africa (SLATE I) (N = 298)	South Africa (SLATE II) (N = 296)
	n (%)	n (%)	n (%)
**Screened for TB**	240 (100)	298 (100)	296 (100)
**Currently on TB treatment***	4 (2)	2 (1)	0 (0)
**1 or more TB symptoms**	90 (38)	105 (35)	140 (47)
**Symptoms reported (among patients with 1 or more symptoms)**			
Cough (current)	75 (83)	71 (68)	76 (54)
Fever	53 (59)	45 (43)	20 (14)
Night sweats	56 (62)	44 (42)	29 (21)
Weight loss	72 (80)	76 (72)	96 (69)
**Number of symptoms reported (n)**			
1 symptom	13 (14)	36 (34)	89 (64)
2 symptoms	18 (20)	27 (26)	32 (23)
3 symptoms	29 (32)	22 (21)	8 (6)
4 symptoms	30 (33)	20 (19)	11 (8)
**TB test performed in symptomatic patients**	63 (70)	60 (57)	111 (79)
**Positive TB tests among symptomatic patients**	12 (13)	9 (9)	6 (5)
**Symptoms among those testing positive**			
Cough (current)	12 (100)	9 (100)	6 (86)
Fever	9 (75)	7 (78)	4 (57)
Night sweats	12 (100)	7 (78)	4 (57)
Weight loss	10 (83)	9 (100)	5 (71)

*Excluded from analyses

For peer review only - http://bmjopen.bmj.com/site/about/guidelines.xhtml

Figure 1. TB testing flow chart at study enrollment among 240 SLATE I intervention arm participants Kenya

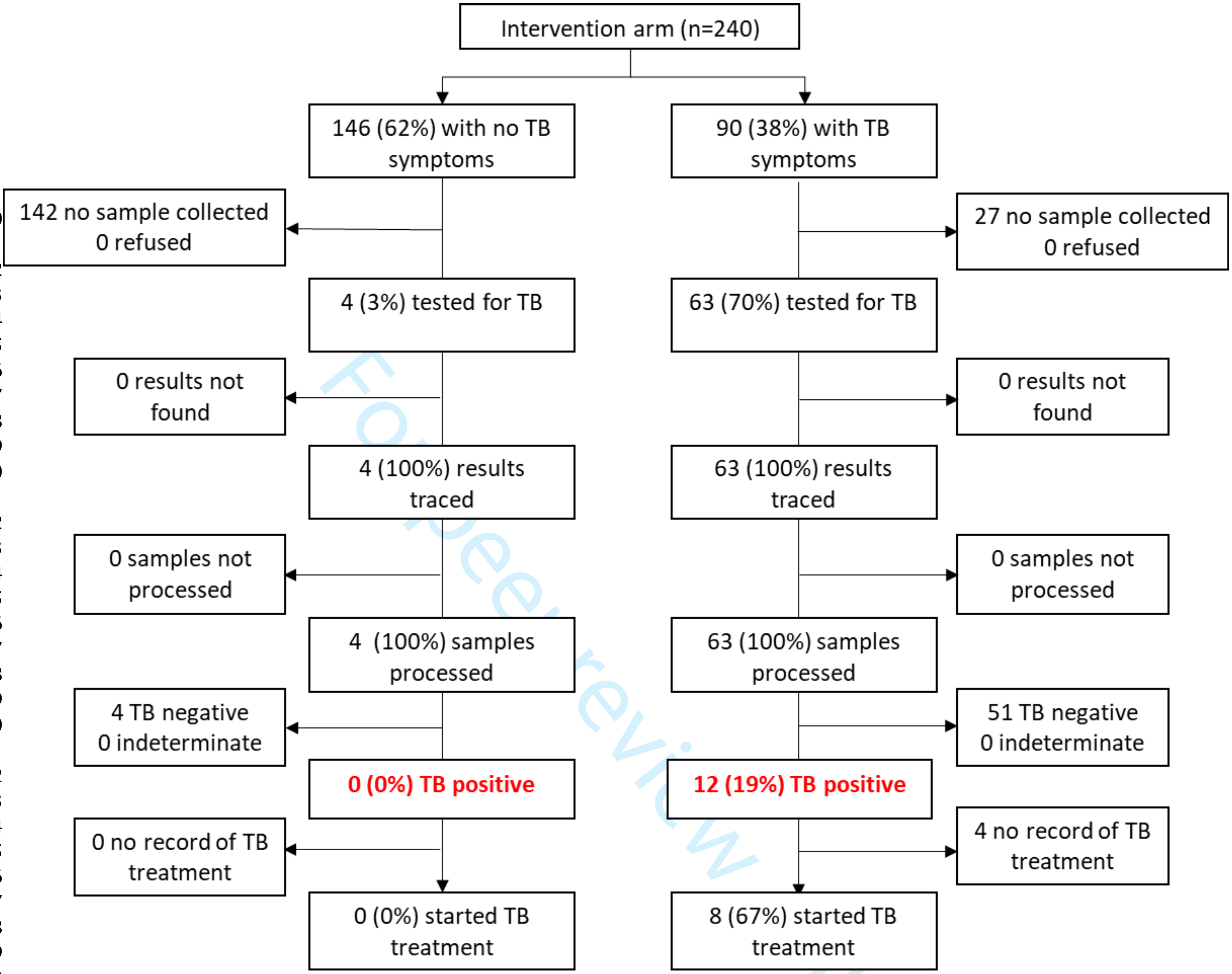

For peer review only - http://bmjopen.bmj.com/site/about/guidelines.xhtml

Figure 2. TB testing flow chart at study enrollment among 298 SLATE I intervention arm participants South Africa

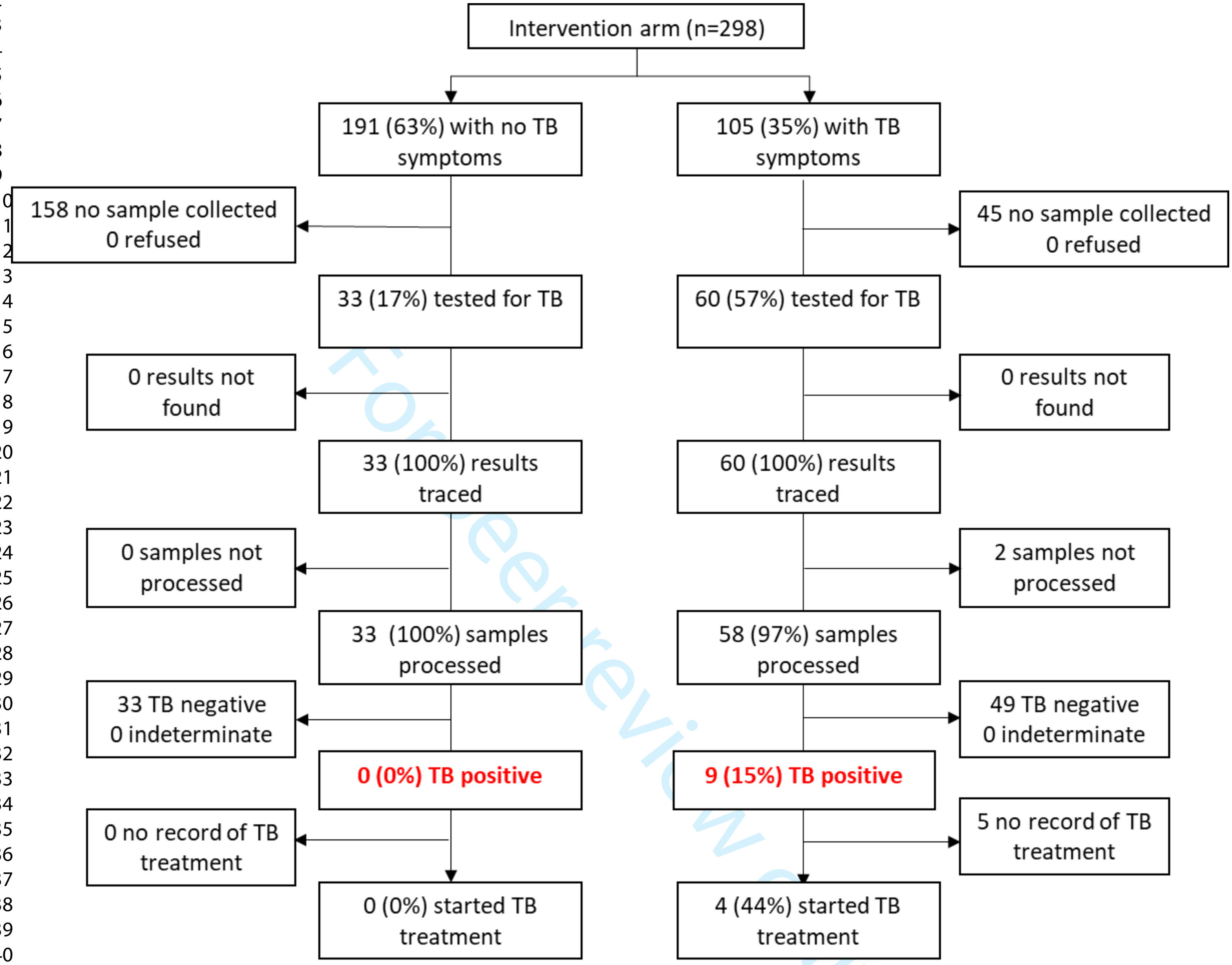

For peer review only - http://bmjopen.bmj.com/site/about/guidelines.xhtml

Figure 3. TB testing flow chart at study enrollment among 296 SLATE II intervention arm participants South Africa

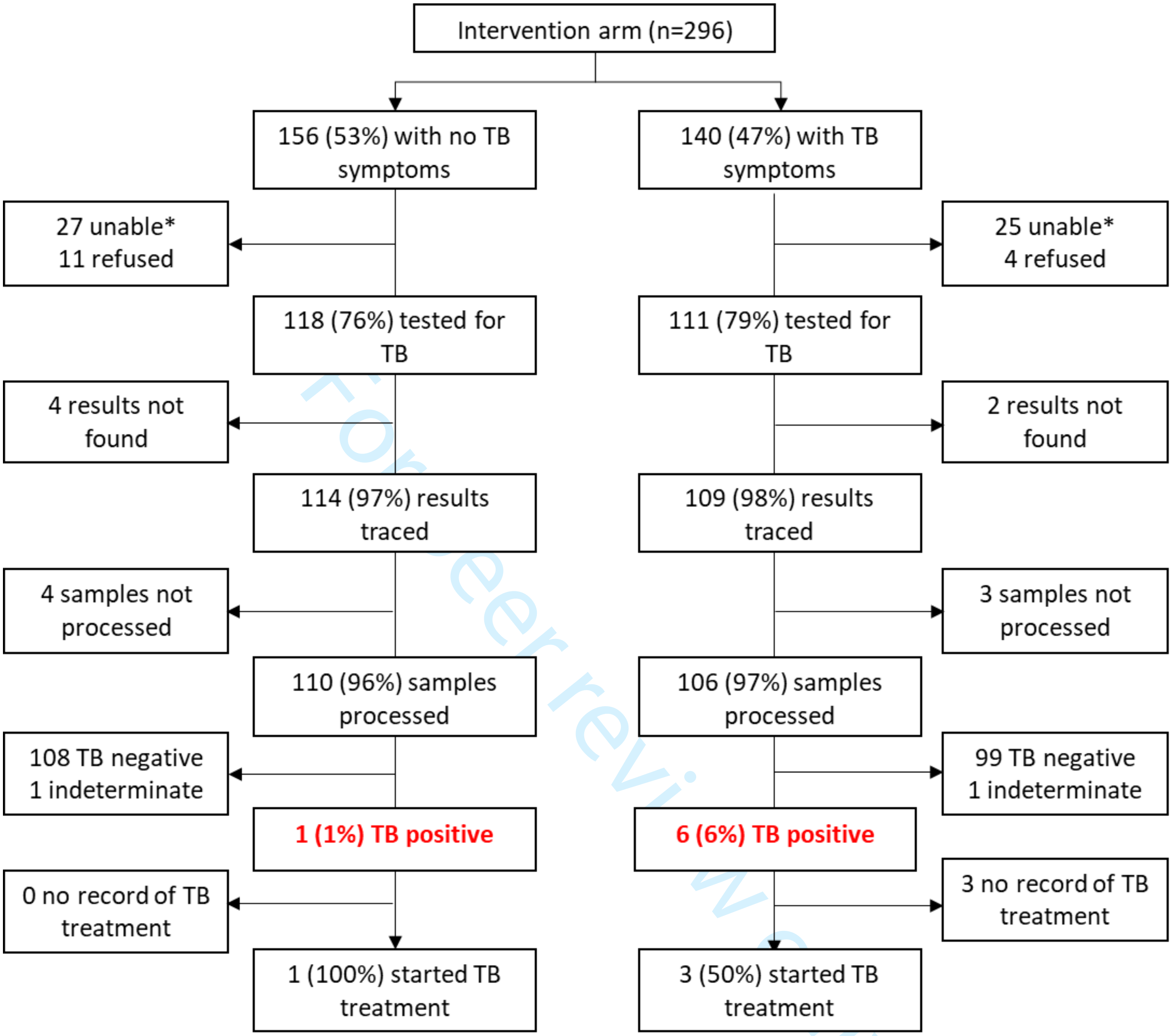

*unable to collect a sputum sample from patient

For peer review only - http://bmjopen.bmj.com/site/about/guidelines.xhtml

Figure 4. Sensitivity, specificity and positive predictive value (with 95% confidence intervals) for the WHO four symptom screen using SLATE II data.

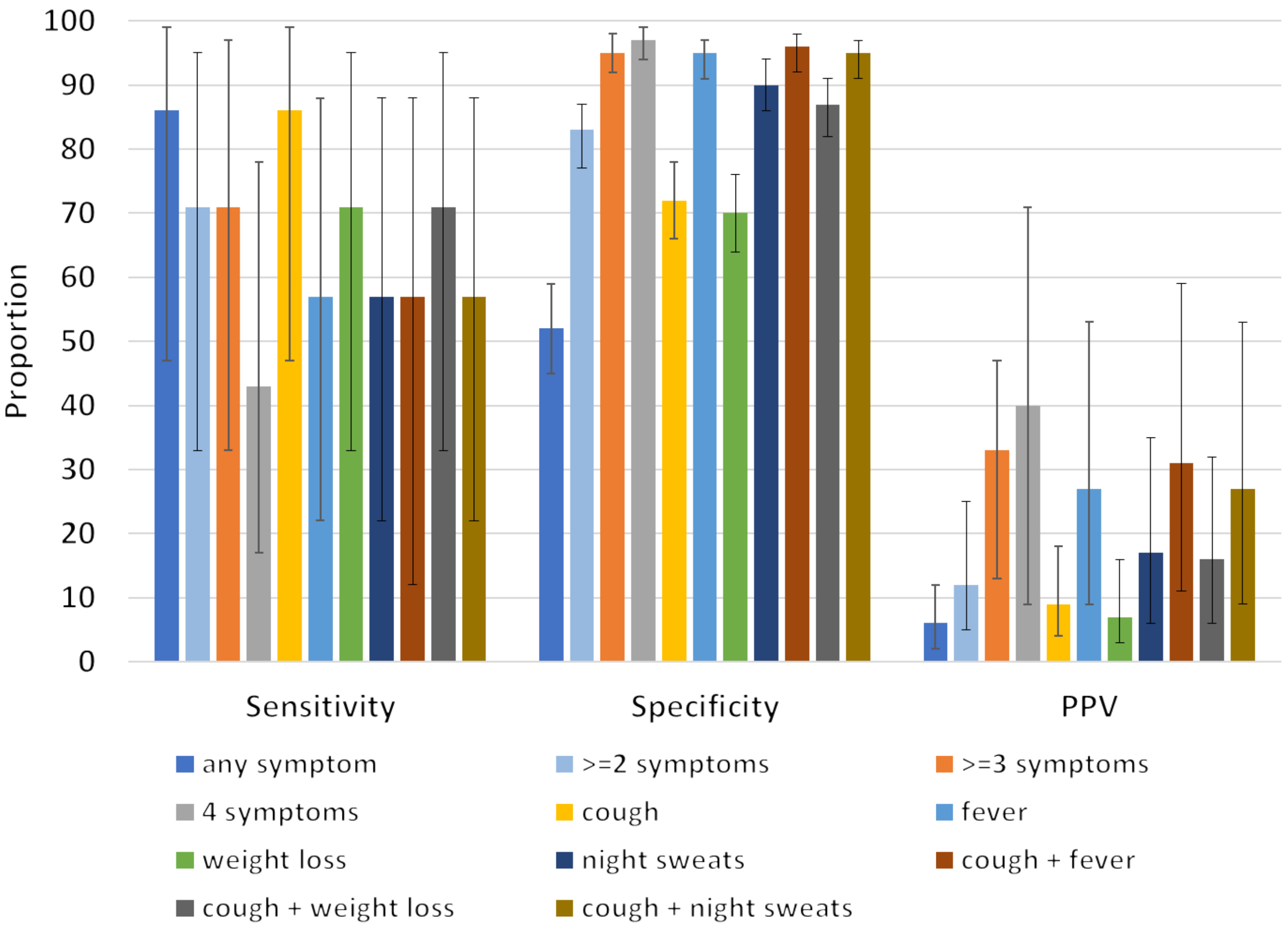

*Negative predictive values are not shown as they remain consistent at above 98% for all analyses.

For peer review only - http://bmjopen.bmj.com/site/about/guidelines.xhtml

Supplementary Fig 1. SLATE I algorithm to support same-day HIV treatment initiation [7]

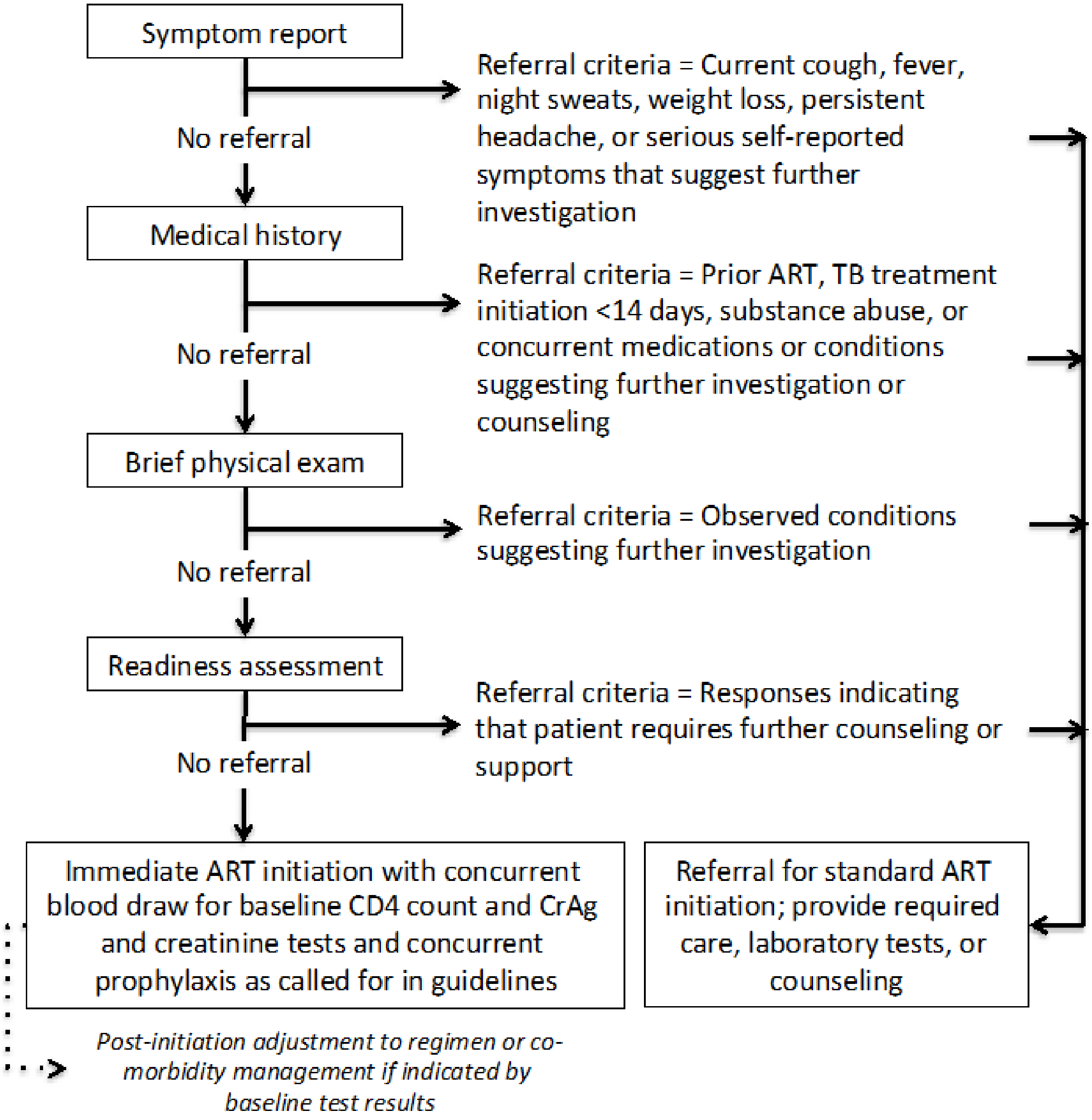

Supplementary Fig 2. SLATE II algorithm to support same-day HIV treatment initiation [8]

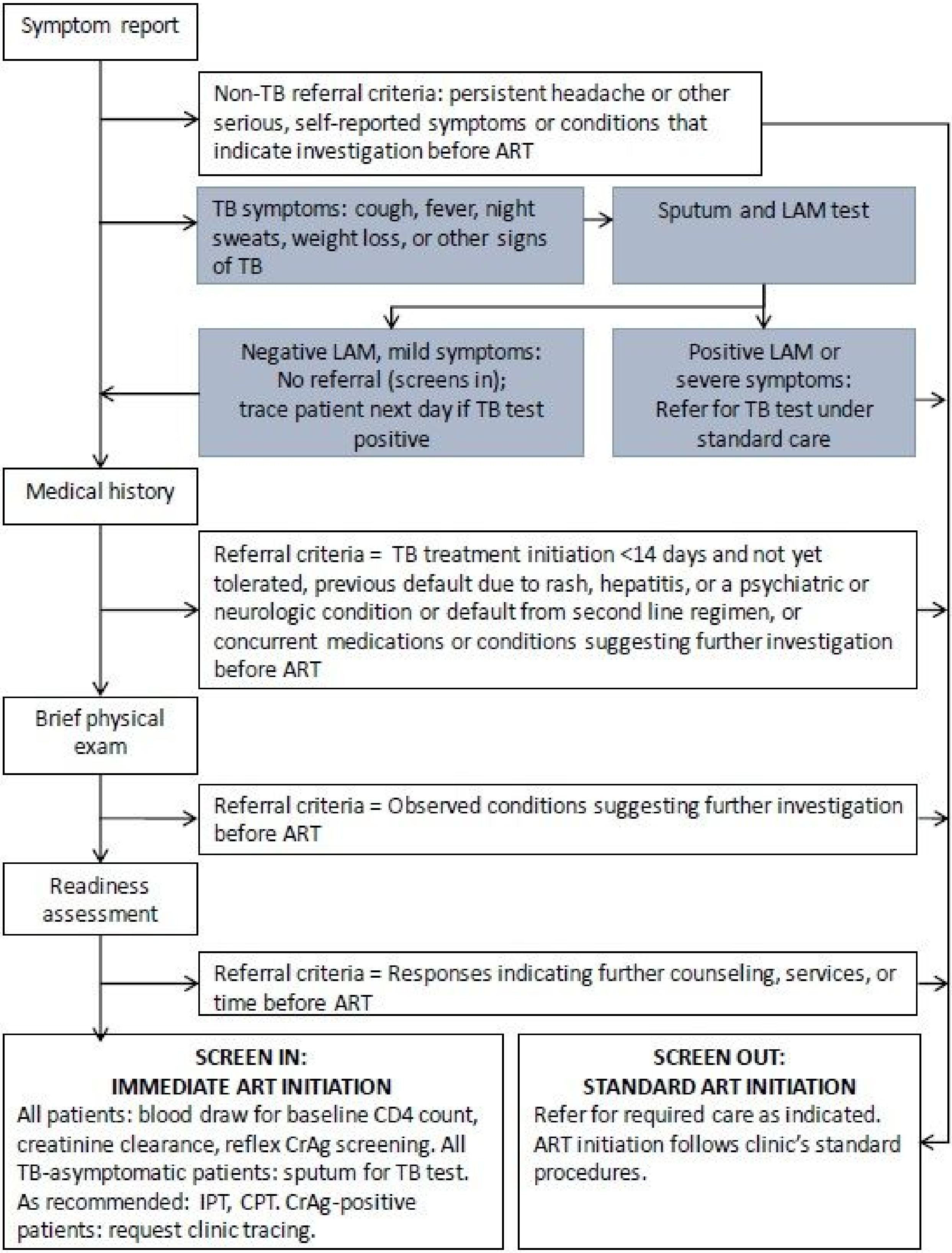

Supplementary Table 1. Characteristics of patients at enrollment in Kenya and South Africa SLATE I and SLATE II trials

Variables†	Categories	Intervention arm Kenya (SLATE I) (N = 240)	Intervention arm South Africa (SLATE I) (N = 298)	Intervention arm South Africa (SLATE II) (N = 296)
		n (%)	n (%)	n (%)
Period of enrollment		July 13, 2017-April 27, 2018	March 6, 2017-July 28, 2017	March 14-Sept 18, 2018
Sex	Female	142 (59)	189 (63)	189 (64)
Age	Median (IQR)	36 (29, 44)	34 (29, 41)	35 (29, 41)
CD4 count at enrollment (cells/mm^3)	Median (IQR)	272 (124, 522)	275 (132, 459)	294 (135, 464)
Location of current residence	Town (urban)	35 (15)	18 (6)	31 (10)
	Peri-urban	66 (28)	299 (89)	265 (90)
	Rural home or village	139 (58	14 (5)	0 (0)
Current house is primary residence	Yes	154 (64)	141 (47)	128 (43)
Number other persons in house	Median (IQR)	3 (2, 5)	2 (1, 3)	1 (1, 3)
Usual activity when well	Formal employment	18 (8)	82 (28)	69 (23)
	Informal sector work	161 (67)	80 (27)	70 (24)
	Unemployed, looking for work	12 (5)	107 (36)	138 (47)
	Other	9 (4)	29 (10)	12 (4)
Transport mode to clinic today (multiple choices possible)	Minibus or taxi	118 (49)	122 (41)	110 (37)
	Walking	46 (19)	156 (53)	161 (54)
	Private car	11 (5)	19 (19)	25 (8)
	Motorbike	137 (57)	0 (0)	0 (0)

For peer review only

Supplementary Table 2. Median CD4 count (cells/mm^3) for patients with and without TB symptoms and those with TB diagnosis in SLATE I and SLATE II trials

Variable	Median CD4 Count Intervention arm Kenya (SLATE I)		Median CD4 Count Intervention arm South Africa (SLATE I)		Median CD4 Count Intervention arm South Africa (SLATE II)	
	n (%)	Median (IQR)	n (%)	Median (IQR)	n (%)	Median (IQR)
**No TB symptoms**	148 (62)	357 (191-632)	191 (63)	307 (141-486)	156 (53)	405 (210-547)
**TB symptom positive**	90 (38)	152 (64-329)	105 (37)	245 (125-425)	140 (47)	175 (89-365)
**TB symptom presence**						
Cough (current)	75 (83)	144 (58-319)	71 (68)	274 (132-433)	76 (54)	157 (68-416)
Fever	53 (59)	136 (49-316)	45 (43)	230 (78-368)	20 (14)	110 (73-203)
Night sweats	56 (62)	215 (56-388)	44 (42)	229 (176-368)	29 (21)	135 (101-367)
Weight loss	72 (80)	146 (53-319)	76 (72)	251 (133-433)	96 (69)	157 (68-337)
**TB symptoms**						
1 symptom	13 (14)	207 (147-317)	36 (34)	188 (72-295)	89 (64)	250 (98-369)
2 symptoms	18 (20)	95 (64-232)	27 (26)	373 (227-520)	32 (23)	137 (76-342)
3 symptoms	29 (32)	232 (72-406)	22 (21)	216 (76-368)	8 (6)	165 (63-350)
4 symptoms	30 (33)	116 (24-277)	20 (19)	267 (180-423)	11 (8)	106 (68-157)
**Symptom prevalence in those diagnosed with TB**	**n=12**		**n=9**		**n=7**	
Cough (current)	12 (100)	124 (12-150)	9 (100)	251 (47-288)	6 (86)	92 (60-107)
Fever	9 (75)	114 (12-150)	7 (78)	201 (18-339)	4 (57)	92 (69-107)
Night sweats	12 (100)	142 (55-224)	7 (78)	201 (18-288)	4 (57)	69 (40-92)
Weight loss	10 (83)	124 (12-150)	9 (100)	251 (47-288)	5 (71)	78 (60-106)

For peer review only - http://bmjopen.bmj.com/site/about/guidelines.xhtml

BMJ Open

Prevalence of TB symptoms, diagnosis and treatment among people living with HIV (PLHIV) not on ART presenting at outpatient clinics in South Africa and Kenya: baseline results from a clinical trial

Journal:	*BMJ Open*
Manuscript ID	bmjopen-2019-035794.R1
Article Type:	Original research
Date Submitted by the Author:	22-May-2020
Complete List of Authors:	Brennan, Alana; Boston University, Departments of Global Health & Epidemiology; Health Economics and Epidemiology Research Office, Department of Internal Medicine, School of Clinical Medicine, Faculty of Health Sciences, University of the Witwatersrand<br>Maskew, Mhairi; Health Economics and Epidemiology Research Office, Department of Internal Medicine, School of Clinical Medicine, Faculty of Health Sciences, University of the Witwatersrand<br>Larson, Bruce; Boston University, Department of Global Health<br>Tsikhutsu, Isaac; Kenya Medical Research Institute; Henry M. Jackson Foundation Medical Research International<br>Bii, Margaret; Kenya Medical Research Institute; Henry M. Jackson Foundation Medical Research International<br>Vezi, Lungisile; Health Economics and Epidemiology Research Office, Department of Internal Medicine, School of Clinical Medicine, Faculty of Health Sciences, University of the Witwatersrand<br>Fox, Matthew; Boston University, Departments of Global Health & Epidemiology; Health Economics and Epidemiology Research Office, Department of Internal Medicine, School of Clinical Medicine, Faculty of Health Sciences, University of the Witwatersrand<br>Venter, Willem Daniel Francois; University of the Witwatersrand, Wits RHI<br>Ehrenkranz, Peter; Bill and Melinda Gates Foundation<br>Rosen, Sydney; Boston University, Department of Global Health; Health Economics and Epidemiology Research Office, Department of Internal Medicine, School of Clinical Medicine, Faculty of Health Sciences, University of the Witwatersrand
**Primary Subject Heading**:	HIV/AIDS
Secondary Subject Heading:	Epidemiology
Keywords:	Tuberculosis < INFECTIOUS DISEASES, HIV & AIDS < INFECTIOUS DISEASES, Clinical trials < THERAPEUTICS

For peer review only - http://bmjopen.bmj.com/site/about/guidelines.xhtml

SCHOLARONE™
Manuscripts

I, the Submitting Author has the right to grant and does grant on behalf of all authors of the Work (as defined in the below author licence), an exclusive licence and/or a non-exclusive licence for contributions from authors who are: i) UK Crown employees; ii) where BMJ has agreed a CC-BY licence shall apply, and/or iii) in accordance with the terms applicable for US Federal Government officers or employees acting as part of their official duties; on a worldwide, perpetual, irrevocable, royalty-free basis to BMJ Publishing Group Ltd ("BMJ") its licensees and where the relevant Journal is co-owned by BMJ to the co-owners of the Journal, to publish the Work in this journal and any other BMJ products and to exploit all rights, as set out in our licence.

The Submitting Author accepts and understands that any supply made under these terms is made by BMJ to the Submitting Author unless you are acting as an employee on behalf of your employer or a postgraduate student of an affiliated institution which is paying any applicable article publishing charge ("APC") for Open Access articles. Where the Submitting Author wishes to make the Work available on an Open Access basis (and intends to pay the relevant APC), the terms of reuse of such Open Access shall be governed by a Creative Commons licence – details of these licences and which Creative Commons licence will apply to this Work are set out in our licence referred to above.

Other than as permitted in any relevant BMJ Author's Self Archiving Policies, I confirm this Work has not been accepted for publication elsewhere, is not being considered for publication elsewhere and does not duplicate material already published. I confirm all authors consent to publication of this Work and authorise the granting of this licence.

For peer review only

Prevalence of TB symptoms, diagnosis and treatment among people living with HIV (PLHIV) not on ART presenting at outpatient clinics in South Africa and Kenya: baseline results from a clinical trial

Short title: TB symptoms at ART initiation

Alana T Brennan[1,2,3*], Mhairi Maskew[3*], Bruce A Larson[1§*], Isaac Tsikhutsu[4,5], Margaret Bii[4,5], Lungisile Vezi[3], Matthew P Fox[1,2,3], Willem DF Venter[6], Peter Ehrenkranz[7], Sydney Rosen[1,3*]

1. Department of Global Health, Boston University School of Public Health, Boston, Massachusetts, United States of America
2. Department of Epidemiology, Boston University School of Public Health, Boston, Massachusetts, United States of America
3. Health Economics and Epidemiology Research Office, Department of Internal Medicine, School of Clinical Medicine, Faculty of Health Sciences, University of the Witwatersrand, Johannesburg, South Africa
4. Kenya Medical Research Institute, Kericho, Kenya
5. Henry M. Jackson Foundation Medical Research International, Inc., Nairobi, Kenya
6. Ezintsha, Wits Reproductive Health and HIV Institute, Department of Internal Medicine, School of Clinical Medicine, Faculty of Health Sciences, University of the Witwatersrand, Johannesburg, South Africa
7. Bill & Melinda Gates Foundation, Seattle, Washington, United States of America

*Corresponding author: Alana Brennan, abrennan@bu.edu

Abstract word count: 299
Manuscript word count: 4598

For peer review only - http://bmjopen.bmj.com/site/about/guidelines.xhtml

Abstract

Objective: We used screening data and routine clinic records for intervention arm patients in the Simplified Algorithm for Treatment Eligibility (SLATE) trials to describe the prevalence of tuberculosis (TB) symptoms, diagnosis and treatment among people living with HIV (PLHIV), not on antiretroviral therapy (ART) and presenting at outpatient clinics in South Africa and Kenya. We compared the performance of the World Health Organization (WHO) four-symptom TB screening tool with a baseline Xpert® test.

Setting: Outpatient HIV clinics in South Africa and Kenya.

Participants: Eligible patients were non-pregnant, PLHIV, ≥18 years of age, not on ART, willing to provide written informed consent. A total 594 patients in South Africa and 240 in Kenya were eligible.

Results: Prevalence of any TB symptom was 38% in Kenya, and 35% (SLATE I) and 47% (SLATE II) in South Africa. During SLATE I, 70% of patients in Kenya and 57% in South Africa with ≥1 TB symptom were tested for TB. In SLATE II, 79% of patients with ≥1 TB symptom were tested. Of those, 19% tested positive for TB in Kenya, and 15% (SLATE I) and 5% (SLATE II) tested positive in South Africa. Of the 28 patients who tested positive in both trials, 20 initiated TB treatment. The lowest median CD4 counts were among those with active TB (Kenya 124cells/mm^3; South Africa 193cells/mm^3). When comparing the WHO four-symptom screening tool to the Xpert® test (SLATE II) we found that increasing the number of symptoms required for a positive screen from 1 to 3 or 4 decreased sensitivity but increased the positive predictive value to >30%.

Conclusions: 80% of patients assessed for ART initiation presented with ≥1 TB symptoms. Reconsideration of the "any symptom" rule may be appropriate, with ART initiation among patients with fewer/milder symptoms commencing while TB test results are pending.

Keywords: Tuberculosis, HIV, Antiretroviral Therapy, Randomized trial, South Africa, Kenya

For peer review only - http://bmjopen.bmj.com/site/about/guidelines.xhtml

Strengths and limitations of this study:

- The data for the analysis presented here come from the intervention arms of two randomized clinical trials (SLATE I in South Africa and Kenya and SLATE II in South Africa), which were conducted at typical primary healthcare clinics in South Africa and typical hospital-based HIV clinics in Kenya.
- The studies enrolled adults not yet on ART, including those just diagnosed and those who had already received some pre-ART care, and collected data at study enrollment about TB symptoms, TB testing, and TB test results.
- We describe types and numbers of symptoms among those with and without TB and compare the performance of symptom screening to laboratory TB test results.
- Limitations of the analysis include heavy reliance on routinely collected data of TB tests performed and their results, likely leading to some missing and incomplete data, and the geographic clustering of the sites in each country, limiting geographic generalizability.

For peer review only

For peer review only - http://bmjopen.bmj.com/site/about/guidelines.xhtml

INTRODUCTION

In 2017 the WHO began recommending rapid ART initiation, including same-day ART initiation (SDI), after the results of several studies indicated that it could reduce loss to follow-up in the pre-ART period[1-3]. The possibility of co-infection with TB, however, remains a major reason for delaying ART among those with the TB symptoms on the WHO four-symptom TB screen (cough, weight loss, fever, night sweats). This symptom screen has been shown to have good sensitivity (89%) but poor specificity (28%) in ART naïve PLHIV[4]. According to the WHO and national guidelines in both South Africa[5] and Kenya[6], patients who report ≥1 TB symptoms require further investigation for active TB disease before ART initiation, which entails a laboratory test such as Xpert® Mycobacterium TB complex /rifampin (MTB/RIF). Following the TB test, patients with negative results resume regular procedures for ART initiation, while those found to have TB are started on TB therapy, with ART initiation delayed until patients are regarded as stable on TB treatment. As a second clinic visit is typically required to receive TB test results, SDI may be impossible for patients presenting with TB symptoms.

The SLATE I study in South Africa and Kenya[7] and the SLATE II study in South Africa[8] evaluated a clinical algorithm to assess eligibility for rapid ART initiation in patients presenting for HIV care but not currently on ART. The algorithms distinguished between patients eligible for SDI of ART and patients requiring referral to clinic staff for additional standard of care evaluation and TB treatment before ART initiation. One or more symptoms of TB—cough, fever, weight loss or night sweats—of any duration or intensity were among the criteria for referral in SLATE I. SLATE II revised the SLATE I algorithm to allow patients with mild TB symptoms and a negative lipoarabinomannan assay (LAM) test[9] to initiate on the same day, based on clinician judgement.

The purpose of this analysis is to describe the prevalence of TB symptoms among patients presenting for HIV care but not currently on ART and to estimate rates of TB testing, diagnosis, and treatment using baseline screening data and routine clinic records for intervention arm patients in the SLATE I and II clinical trials in South Africa and Kenya. We also assessed the performance of the WHO four-

For peer review only - http://bmjopen.bmj.com/site/about/guidelines.xhtml

symptom TB screening tool compared to Xpert® MTB/RIF sputum testing. The implications of current TB screening, diagnosis, and treatment for implementation of SDI are discussed.

METHODS

SLATE trials

The SLATE I and SLATE II trials in South Africa and Kenya were multicenter trials evaluating two variations of a simplified algorithm to determine eligibility for SDI of ART without relying on laboratory results or multiple clinic visits[7, 8]. Enrollment for SLATE I (NCT02891135) was completed in July 2017 in South Africa and April 2018 in Kenya. Enrollment for SLATE II (NCT03315013), only conducted in South Africa, was completed in September 2018.

Study population

Patient inclusion and exclusion criteria for study eligibility have been described previously[7,8]. Briefly, those enrolled were non-pregnant, PLHIV, ≥18 years of age, not currently on ART and willing to provide written informed consent, who were randomized 1:1 to the intervention and standard of care arms. This analysis is limited to patients randomized to the intervention arm, for whom we have TB symptom data. Symptom screening of patients in the standard of care arm was poorly documented by clinic staff. Intervention arm patients were assessed for eligibility for SDI by a study nurse (South Africa) or clinical officer (Kenya) using the SLATE I or II algorithm, which each consisted of four screens: (1) current symptoms, (2) recent medical history, (3) physical conditions, and (4) treatment readiness (see Supplementary Figure 1 and Supplementary Figure 2). The baseline characteristics of these patients have previously been described[10].

Approval of the SLATE trials was provided by the Human Research Ethics Committee of the University of the Witwatersrand in South Africa (SLATE I 160910; SLATE II 171011), the Kenya Medical Research Institute (SLATE I 3408) and the Walter Reed Army Institute of Research in Kenya (SLATE I 2401) , and the Institutional Review Board of Boston University (SLATE I H-35634; SLATE II H-37010).

Patient and Public Involvement

For peer review only - http://bmjopen.bmj.com/site/about/guidelines.xhtml

Patients were not involved in the design, recruitment or conduct of the SLATE trials. We conducted a qualitative study at the end of SLATE II to help gain a deeper understanding of patient perceptions of initiating ART per standard of care compared to same-day initiation using the SLATE algorithm. Presentation of primary study results will be conducted by study staff at participating clinics to disseminate research findings to staff and patients prior to funding ending in July 2020.

TB screening and diagnosis differences between SLATE I and SLATE II

The main difference between the algorithm used in SLATE I and the revised version used in SLATE II was the approach to TB screening and diagnosis. Both algorithms asked patients for self-reported TB symptoms (any cough, fever, weight loss, or night sweats of any duration), based on the WHO four-symptom screen[11]. Procedures differed from that point forward, as described below and illustrated in supplementary figures 1 and 2.

In SLATE I, consistent with national guidelines in both countries, patients reporting any symptoms of TB of any severity or duration were referred to routine clinic care, which should have included a TB test according to guidelines. Each patient was given a referral letter for the clinic indicating the reason for referral, such as the presence of TB symptoms, but no further effort was made by study staff to ensure that the patient remained in care or was tested or treated for TB.

As has previously been reported[12], in SLATE I and illustrated in Figures 1 and 2, a larger proportion of intervention arm patients than expected were ineligible for SDI due to TB symptoms (38% in Kenya and 37% in South Africa). The presence of $\geq$1 more TB symptoms was by far the main reason for ineligibility under the SLATE I algorithm. Very few of these patients were ultimately confirmed to have TB, however, and study patients reported no TB-related adverse events after starting ART. We thus speculated that referring a patient out for mild TB symptoms, without further complications, was too stringent a requirement, and we developed the SLATE II algorithm accordingly.

In SLATE II, if a patient reported $\geq$1 TB symptoms, the newly developed TB module in the algorithm was applied. The TB module included: (1) a more detailed medical history (e.g. inquiring about severity and

For peer review only - http://bmjopen.bmj.com/site/about/guidelines.xhtml

duration of symptoms); (2) a focused physical examination to assess TB symptoms; (3) sputum collection for Xpert® MTB/RIF sputum testing; and (4) a point-of-care, urine-based LAM test (Determine TB LAM Ag, Abbott, Waltham, MA, USA)[9]. Patients with a positive LAM test, TB symptoms that were severe or of long duration, or any other clinical finding indicating active TB were referred back to routine care under the SLATE II algorithm. As with SLATE I, no effort was made by study staff to ensure that the patient underwent a TB test once referred back to routine care. A patient with a negative LAM test and without severe symptoms of TB remained eligible for same day initiation of ART. For SLATE II, unlike SLATE I, the study staff collected a sputum sample from all intervention arm patients who were able to produce one, regardless of TB symptoms. These samples were sent to the National Health Laboratory Service for Xpert® MTB/RIF sputum testing for TB disease.

Data collection

We collected demographic and algorithm data for intervention arm patients via a case report form administered at study enrollment. Laboratory tests results (e.g., CD4 counts and Xpert® MTB/RIF findings) were extracted directly from laboratory electronic records or paper-based registers kept at each site, while follow-up data for the study period were collected from routinely generated clinical record data from patient records in electronic and paper format.

Statistical analysis

Simple descriptive statistics were used to display demographic and clinical characteristics of patients at study enrollment, prevalence of TB symptoms, TB diagnosis and TB treatment.

Having Xpert® MTB/RIF sputum tests on the majority (n=214) of SLATE II intervention arm patients provided a unique opportunity to assess the performance of the WHO four-symptom TB screening tool. We defined 11 possible interpretations of TB symptom screening results and compared them to the gold standard of Xpert® MTB/RIF: 1) any TB symptom; 2) $\geq$2 symptoms; 3) $\geq$3 TB symptoms; 4) all 4 TB symptoms; 5) cough alone; 6) fever alone; 7) weight loss alone; 8) night sweats alone; 9) cough and fever; 10) cough and night sweats and 11) cough and weight loss. We calculated the sensitivity (probability of screening positive (using each definition above) when TB disease is present as defined

For peer review only - http://bmjopen.bmj.com/site/about/guidelines.xhtml
For peer review only

by Xpert® MTB/RIF), specificity (probability of screening negative when TB disease is not present as defined by Xpert® MTB/RIF), positive predictive value (probability of a patient having TB disease when the screen is positive) and negative predictive value (the probability of a patient not having TB disease when the screen is negative).

RESULTS

Demographic and clinical characteristics

Supplementary Table 1 summarizes basic characteristics of intervention arm patients stratified by trial cohort (N=240 for SLATE I Kenya; N=298 for SLATE I South Africa; N=296 for SLATE II). The majority of patients in each group were females in their mid-thirties, with a median baseline CD4 count between 272 and 294 cells/mm^3. We excluded 4 patients in Kenya and 1 in South Africa in the SLATE I trial who were known to be on TB treatment at study enrollment (Table 1). Further details of baseline characteristics of the cohorts have been reported previously[10].

TB symptom prevalence

The prevalence of any TB symptoms was 38% [95% confidence interval:32-44%] in SLATE I in Kenya, 35% [30-41%] in SLATE I in South Africa, and 47% [42-53%] in SLATE II (Table 1). In both studies and both countries, amongst people with any symptom, cough (66% combined) and weight loss (72% combined) were the most common symptoms reported. As presented in Supplementary Table 2, patients with TB symptoms had substantially lower CD4 counts in all three cohorts at study enrollment than did those with no symptoms of TB, indicating more advanced HIV disease among symptomatic patients. We saw little variation in CD4 count when the data were stratified by number of symptoms or symptom type. The lowest median CD4 counts were recorded among those found to have active TB disease (Kenya 124 cells/mm^3 [12-150]; South Africa (SLATE I and II combined) 193 cells/mm^3 [56-223].

TB testing and diagnosis among symptomatic patients

In Kenya (SLATE I), 90 (38% [32-44%]) intervention arm patients had $\geq$ 1 symptom of TB and screened out of the algorithm, with referral back to standard care. Clinic staff chose or were able to test only 63/90 (70% [60-79%]) symptomatic patients for TB. Of those tested, 12/63 (19% [11-30%]) had a

For peer review only - http://bmjopen.bmj.com/site/about/guidelines.xhtml

positive result, corresponding to an estimated TB prevalence in symptomatic patients presenting for care in Kenya of 5% [3-8%] (assuming those not tested were negative for TB disease) (Figure 1). In SLATE I in South Africa, we saw a similar proportion of patients presenting with at least 1 symptom of TB (105, 35% [30-41%]), but only 60 (57% [48-66%]) of these symptomatic patients were tested for TB by the study clinics. Of those tested, 9/60 (15% [8-26%]) had a positive result, corresponding to an estimated TB prevalence in patients presenting for care in South Africa during SLATE I of 3% [2-5%] (Figure 2), with the same assumption regarding the negative status of those not tested.

In SLATE II, all 296 intervention arm patients were asked for a sputum sample per the study protocol[8], regardless of symptoms. We were able to successfully collect and test sputum for 111 (79% [72-85%]) symptomatic patients and 118 (76% [68-82%]) asymptomatic patients, or 72% (n=214/296) overall (Figure 3). Of the 29 symptomatic patients not tested, 25 were unable to produce a sputum sample and 4 refused to test. Among the 106 symptomatic patients in SLATE II with a successful test, 6 (5% [2-11%]) results were positive for TB, producing an estimated TB prevalence in all PLHIV (asymptomatic and symptomatic) presenting for care in South Africa during SLATE II of 2% [1-5%], with the same assumption regarding the negative status of those not tested.

Amongst the total of 28 TB-positive patients in both studies, 92% of patients in Kenya and 81% in South Africa (SLATE I and II combined) had at least three symptoms of the disease, and 67% in Kenya and 63% in South Africa had all four symptoms. Virtually all (96% [84-99%]) those diagnosed presented with a cough. Weight loss was also common (86% [69-95%]), followed by night sweats (82% [65-93%]) and fever (71% [53-86%]) (Table 1).

TB testing and diagnosis in asymptomatic patients

Among the 337 (63% [58-67%]) patients with no TB symptoms in SLATE I, four (3% [1-6%]) patients in Kenya and 33 (17% [12-23%]) patients in South Africa were tested for TB by the study clinics. In Kenya, three of the patients had documented reasons for being tested by the clinic (one was taking cough syrup for the previous 5 days and was recently screened for TB, one had suspected extra-pulmonary TB due to swelling in the jaw, and one patient was asthmatic). The remaining patient had no documented

For peer review only

For peer review only - http://bmjopen.bmj.com/site/about/guidelines.xhtml

reason for a TB test. In South Africa, one of the clinics in our study was attempting to collect sputum from all PLHIV prior to ART initiation, regardless of symptoms. None of the 33 asymptomatic patients tested were positive (Figures 1 and 2).

In SLATE II, as per the study protocol[8], all 296 intervention arm patients were asked for a sputum sample. We were able to successfully collect and test sputum for 118 (76% [68-93%]) asymptomatic patients (Figure 3). One positive TB test result was recorded among those who were eligible for SDI under the SLATE II algorithm. This was an asymptomatic patient who had a negative LAM test and CD4 count of 350 cells/mm^3. The patient was successfully traced and commenced TB treatment the following day.

TB LAM test results

In SLATE II, all intervention arm subjects with ≥1 symptoms of TB had a LAM test performed. Only two tests (<1%) were positive. Both patients had sputum samples taken for Xpert® testing. The first patient was a male who came to the clinic for care because he felt unwell. He reported all four TB symptoms and had a CD4 count of 78 cells/mm^3. This patient's result came back positive for TB and he was successfully traced and commenced on TB treatment. The second patient was a female who came for an HIV test and reported cough and weight loss and had a CD4 count of 19 cells/mm^3. This patient tested negative for TB on Xpert®.

TB treatment uptake

In Kenya, eight of the 12 patients (67% [38-88%]) who tested positive for TB went on to initiate treatment for the disease, all within two weeks of diagnosis. Of the remaining four patients, one died within three weeks of study enrollment with no record of starting TB treatment, and the remaining three remained in care but had no record of initiating TB treatment. Of the 16 patients (symptomatic and asymptomatic) who tested positive in SLATE I and II in South Africa, eight initiated TB treatment after diagnosis (four patients within one week and four within seven weeks after study enrolment), four patients remained in care but had no record of starting TB treatment, three patients were

For peer review only - http://bmjopen.bmj.com/site/about/guidelines.xhtml

transferred to another facility before starting TB treatment, and one patient was lost from care before starting treatment.

Unmasking TB disease

One of the major concerns about SDI is immune reconstitution inflammatory syndrome among patients with undiagnosed TB (TB-IRIS). No patients in either study (both standard of care and intervention arms) had indications in their routine clinic records of IRIS reactions during passive study follow up, though this may reflect incomplete record-keeping. Patient clinical records indicated that clinic staff investigated a total of 25 patients for TB more than 30 days after study enrollment. Of these, 24 test results were available (we were unable to locate one test result in SLATE I in South Africa) and two were positive for TB disease (one positive patient was in the standard of care arm in South Africa (SLATE I) and one was in the intervention arm in Kenya (SLATE I) and not eligible for SDI). Among these 24 patients with a follow-up TB test, 4 (17%) (three in SLATE I and one in SLATE II) were eligible for SDI via the SLATE algorithm, none of these patients had a positive TB test.

In the rest of the results section, we will focus solely on SLATE II in South Africa, where the new TB module in the SLATE II algorithm was applied.

TB in symptomatic patients eligible for SDI under SLATE II algorithm

In the SLATE II study, patients with milder TB symptoms and a negative TB LAM test were eligible for SDI under the study at the discretion of the study nurse. Of the intervention arm patients who screened in and were eligible for SDI, 40% [34-46%] (n=101) presented with at least one of the four TB symptoms. Of these, 76% [66-83%] (n=77) reported only one symptom, 20% [13-28%] (n=20) reported two symptoms, and 4% [1-9%] (n=4) reported 3 or more symptoms. The most frequently reported symptoms were weight loss (64%) and cough (45%); night sweats (13%) and fever (6%) were less frequently reported. Of the 101 symptomatic patients, 75% [64-81%] (n=74) successfully completed a TB test (the remainder either could not produce a sputum sample or were unwilling to do so) and of these, only one tested positive for TB. This patient reported only cough and had a negative LAM test during administration of the SLATE II algorithm. After SDI, the patient, whose CD4 count was 116

For peer review only - http://bmjopen.bmj.com/site/about/guidelines.xhtml

cells/mm^3, could not be traced to start TB treatment, despite phone calls and a home visit. The patient never returned to the clinic after study enrolment for either TB or HIV care.

Sensitivity, specificity and predictive values of SLATE II algorithm

Figure 4 displays the results of the performance of the WHO four-symptom screen in SLATE II intervention arm patients. The sensitivity and specificity of any single TB symptom were 86% [47-99%] and 52% [45-59%], respectively. The positive predictive value was 6% [2-12%], while the negative predictive value was 99% [96-99%]. When modifying the definition of a positive TB screen to ≥3 symptoms, sensitivity decreased to 71% [33-95%] and specificity increased to 95% [92-98%]. The positive predictive value improved to 33% [13-59%] while negative predictive value remained unchanged (99% [97-99%]). The WHO symptom screening tool appeared to perform the best for sensitivity (86% [47-99%]) and specificity (72% [66-78%]) when using cough only. However, the positive predictive value was the highest in the analysis using ≥3 (33% [13-59%]) and 4 (40% [14-71%]) symptoms. In all other analyses of the WHO four-symptom screen, sensitivity falls, and specificity increases as a result, while the positive predictive value ranges from 7% to 31% depending on how the prevalence shifts. The negative predictive value remained unchanged for all analyses at above 98%. This was expected as the prevalence of TB in the population was low (3%) based on Xpert® testing.

DISCUSSION

In three cohorts of PLHIV not yet on ART and presenting for care in Kenya and South Africa since 2017, we estimated an overall prevalence of any TB symptoms of 35% to 50%. Between 60% and 80% of these patients were tested for TB, among whom 19% in Kenya and 6% to 15% in South Africa had a positive TB test using Xpert MTB/RIF, corresponding to an estimated prevalence in patients presenting for care of 5% in Kenya and 2% to 3% in South Africa (assuming those not tested are negative for TB disease). The results we report for TB symptom prevalence (with cough also being the most common symptom reported) and percentage of patients with TB symptoms tested for TB reported in our study are consistent with what has been previously published at a national level in Kenya[13] and in HIV study cohorts in South Africa[14-21]. The consistency of our estimates of TB disease prevalence with

For peer review only - http://bmjopen.bmj.com/site/about/guidelines.xhtml

previously published literature varied. Some studies report estimates that were comparable[13,14], lower[15], or higher[16-21]. National estimates of TB prevalence are still sparse in South Africa. However, the National Tuberculosis Prevalence Survey is currently underway is currently underway in South Africa, so better estimates will be available soon[22].

In our studies, over 80% of the 28 patients who tested positive for TB disease reported having at least three clinical symptoms of the disease, the most common being a cough, followed by weight loss, night sweats, and fever. Other studies have estimated more than 80% of patients diagnosed with TB had more than one symptom of the condition[18,19,23]. A study in South Africa also reported that more than 60% of patients without TB disease had symptoms [23], again consistent with our findings of 59%.

Using SLATE II results, the performance of the WHO four-symptom screen tool, when classifying patients with $\geq$1 symptoms of TB, was comparable regarding sensitivity to what was reported in a recent meta-analysis[4], but we had a higher specificity at 52% (vs. 28%[4]). When we increased the number of symptoms required for a positive screen to 3 or 4, or to just 4, we saw a decrease in sensitivity and subsequent increase in specificity and an increase in the positive predictive value from 6% (for $\geq$1 symptoms of TB) to 33% and 40%, respectively.

When delaying ART in person living with HIV is contingent on a positive TB test, the positive predictive value is more clinically relevant than sensitivity or specificity. The main reason for the delay of ART initiation in TB suspect patients is to prevent TB-IRIS. The estimated risk of TB-IRIS is quite low, however, at roughly 1% to 6% in sub-Saharan Africa[24]. This risk is highest in patients presenting with CD4 counts <100 cells/mm^3[25,26]. The median CD4 count in all three of our cohorts amongst symptomatic patients was above 100 cells/mm^3, while patients presenting with no symptoms had a median CD4 count above 300 cells/mm^3. The lowest median CD4 counts, and those at highest risk of TB-IRIS, were amongst patients who had three if not all four symptoms of TB disease and had a positive Xpert® test. In a healthcare system that can produce TB test results soon after ART initiation and successfully contact (trace) patients with positive results, it may be reasonable to increase the number of TB symptoms required to trigger a delay in ART initiation to 3 or 4. This would increase the

For peer review only - http://bmjopen.bmj.com/site/about/guidelines.xhtml

probability that a patient who is classified as TB positive (using the WHO four-symptom screen) truly has TB disease and allow patients with fewer or milder TB symptoms to start ART while TB test results are pending. It is still unknown whether initiating ART prior to TB being confirmed or treated causes harm (or benefit). In a setting in which TB tests are delayed and/or active tracing of patients with positive results is poor, in contrast, a more conservative approach—delaying ART until a TB test can be completed for patients with even one symptom—may continue to be justified. However, more research is required to accurately weigh the risks of delaying ART (which might prevent a person from being successfully linked into HIV treatment) versus the benefits of waiting for a definitive TB diagnosis to minimize the already low and potentially manageable clinical risk of TB-IRIS.

In both Kenya and South Africa, national TB guidelines during the study stated that PLHIV self-reporting any TB-related symptoms should be tested with Xpert® MTB/RIF and treated if diagnosed with the disease. In SLATE I, we saw gaps in following national guidelines in both countries. Twenty-seven symptomatic, intervention arm patients in Kenya and 45 in South Africa were ineligible for SDI due to TB symptoms and were referred back to the clinic for further testing but were not tested for TB by the clinic staff. We assume that a certain number of patients refused or were unable to provide a sputum sample for testing, but for some, the nurse or clinical officer who saw the patient chose not to do a test. At one study site in South Africa we were told, informally, that staff only requested TB tests if two or more symptoms were present, while at one site in Kenya, a clinical officer would diagnose a respiratory infection before TB and require the patient to go through a course of antibiotics, advising the patient to return for a TB test only if symptoms persisted. Whether failure to follow guidelines precisely reflects reasonable use of clinical judgment, lack of available resources, or irresponsible non-compliance on the part of clinic staff is unclear, but it should be considered in efforts to improve treatment of TB disease and maximize opportunities to offer TB preventative treatment for patients without TB.

The urine TB-LAM test used in SLATE II (the Determine TB LAM Ag, as specified above) was positive in <1% of our intervention arm patients in the SLATE II trial. This test has been shown to improve TB diagnosis in patients with low CD4 counts[27], so the low yield we saw could be due to higher CD4

For peer review only - http://bmjopen.bmj.com/site/about/guidelines.xhtml

counts in our cohort. The average CD4 count in SLATE II intervention arm patients was 294 cells/mm^3, it was 175 cells/mm^3 amongst those with TB symptoms and ranged from 60-107 cells/mm^3 amongst those diagnosed with TB. Also, previous studies have shown that the positive predictive value of urine LAM testing depends upon the prevalence of TB disease in the population, which was low (6%) in our SLATE II cohort[28]. The LAM test used in the study may thus not offer much benefit for the cost involved.

Our study had several limitations. First, while the study sites were all typical primary healthcare clinics in South Africa and typical hospital-based HIV clinics in Kenya, they were geographically clustered in each country, making generalizability to the rest of the country uncertain. Second, we relied heavily on routinely collected data pertaining to TB test conduct and results, and it is likely that some TB tests were ordered but not analyzed or analyzed but not recorded. Similarly, data on events after the study enrollment visit, such as post-initiation TB diagnoses or disease, were likely incomplete, and some events may not have been reported to healthcare facilities at all. Third, we assumed that patients who did not have TB tests were TB-negative; it is possible that some in fact had TB, making prevalence in our population higher than reported. Finally, our sample sizes were small, limiting our ability to stratify by patient characteristics; a larger sample might identify other characteristics associated with a positive TB test. The small sample size could also affect our ability to detect TB-related adverse events in our sample.

Despite these limitations, we conclude that in the SLATE I and SLATE II trials, among 235 patients with WHO-defined TB symptoms who were not eligible for SDI, over 80% did not have TB, making any delay to ART initiation due to the requirement for a pre-initiation TB test unnecessary. No serious, TB-related adverse events were reported after starting ART among symptomatic patients with or without delay in our study. Reconsideration of WHO's guidance to even "briefly" delay ART initiation based on presence of "any TB symptom" may be appropriate, with ART initiation among patients with fewer or milder symptoms commencing while TB test results are still pending.

Acknowledgments

For peer review only - http://bmjopen.bmj.com/site/about/guidelines.xhtml

We thank the patients who participated in the study and the staff of the six study clinics for their cooperation; the City of Johannesburg and Ekhureleni Metro in South Africa and the management of Kericho County Referral Hospital, Kapsabet County Referral Hospital, and Kombewa County Hospital in Kenya for permission to conduct this study.

Contributors: MF, WV and SR had substantial contributions to the conception and design of the work. AB, MM and BL acquisition, analysis or interpretation of data. AB, MM, BL, IT, MB, LV, MF, WV, PE and SR contributed substantially to the drafting and revising the work critically for important intellectual content. All the authors have read and approved the final version of the manuscript. All authors are willing to be accountable for the work and in ensuring that questions related to the accuracy and integrity of the work were appropriately investigated and resolved.

Competing interests: None declared.

Funding: Research was funded by the Bill and Melinda Gates Foundation (Grant No. OPP1136158)

Data sharing statement: Data generated by the study will be made publicly available in the Dryad repository (http://www.datadryad.org/) after the protocol has been closed (anticipated closure July 2020). Until then, data will remain under the supervision of the Boston University Medical Campus IRB, the University of the Witwatersrand Human Research Ethics Committee (HREC), and the KEMRI Scientific and Ethics Research Unit. Requests can be sent to the BUMC IRB at medirb@bu.edu. Data extracted from routine medical records are owned by the study sites, the South African National Department of Health, and the Kenyan Ministry of Health and cannot be made publicly available by the authors.

For peer review only

For peer review only - http://bmjopen.bmj.com/site/about/guidelines.xhtml

References

1. Guidelines for Managing Advanced HIV Disease and Rapid Initiation of Antiretroviral Therapy. Geneva: World Health Organization; 2017.
2. Amanyire G, Semitala FC, Namusobya J, Katuramu R, Kampiire L, Wallenta J et al. Effects of a multicomponent intervention to streamline initiation of antiretroviral therapy in Africa: a stepped-wedge cluster-randomised trial. Lancet HIV. 2016;3:e539–48.
3. Rosen S, Maskew M, Fox MP, Nyoni C, Mongwenyana C, Malete G et al. Initiating antiretroviral therapy for HIV at a patient's first clinic visit: the RapIT Randomized Controlled Trial. PLoS Med. 2016;13:e1002015.
4. Hamada Y, Lujan J, Schenkel K, Ford N, Getahun H. Sensitivity and specificity of WHO's recommended four-symptom screening rule for tuberculosis in people living with HIV: a systematic review and meta-analysis. Lancet HIV. 2018 Sep;5(9):e515-e523.
5. National Department of Health. Same-day antiretroviral therapy (ART) initiation for HIV positive patients. Pretoria: National Department of Health; 2017.
6. Ministry of Health and National AIDS and STI Control Program (NASCOP). Guidelines on the Use of Antiretroviral Drugs for Treating and Preventing HIV Infection in Kenya 2018 Edition. Nairobi, Kenya; 2018.
7. Rosen S, Fox MP, Larson BA, Brennan AT, Maskew M, Tsikhutsu I, Bii M, Ehrenkranz PD, Venter WF. Simplified clinical algorithm for identifying patients eligible for immediate initiation of antiretroviral therapy for HIV (SLATE): protocol for a randomised evaluation. BMJ Open. 2017 May 28;7(5):e016340.
8. Rosen S, Maskew M, Brennan AT, Fox MP, Vezi L, Ehrenkranz PD, Venter WDF. Improved simplified clinical algorithm for identifying patients eligible for immediate initiation of antiretroviral therapy for HIV (SLATE II): protocol for a randomized evaluation. Trials. 2018 Oct 11;19(1):548.
9. World Health Organization. The use of lateral flow urine lipoarabinomannan assay (LF-LAM) for the diagnosis and screening of active tuberculosis in people living with HIV TB. Geneva: World Health Organization, 2015.
10. Brennan A, Maskew M, Larson B, Tsikhutsu I, Bii M, Vezi L, Fox M, Venter F, Ehrenkranz P, Rosen S. Who is seeking antiretroviral treatment for HIV now? Characteristics of patients presenting in Kenya and South Africa in 2017-2018. J Int AIDS Soc; in press.
11. World Health Organization. Systematic screening for active tuberculosis Principles and recommendations. Geneva: World Health Organization, 2013.
12. Maskew M, Larson B, Brennan AT, Fox MP, Vezi L, Tsikhutsu I, Bii M, Venter WDF, Rosen S. Same-day ART initiation without laboratory tests is safe and effective: primary outcomes of the SLATE trial in South Africa and Kenya. PLoS Medicine. 2019.
13. Kenya Tuberculosis Prevalence Survey. Republic of Kenya. Minisrty of Health. 2016. Available at: https://www.chskenya.org/wp-content/uploads/2018/04/Final-TB-Prevalence-Survey-Report.pdf.
14. Rangaka MX, Wilkinson RJ, Glynn JR, Boulle A, van Cutsem G, Goliath R, et al. Effect of antiretroviral therapy on the diagnostic accuracy of symptom screening for intensified tuberculosis case finding in a south African HIV clinic. Clin Infect Dis. 2012;55(12):1698–706.

For peer review only - http://bmjopen.bmj.com/site/about/guidelines.xhtml

15. Hanrahan CF, Nonyane BAS, Mmolawa L, West NS, Siwelana T, Lebina L, Martinson N, Dowdy DW. Contact tracing versus facility-based screening for active TB case finding in rural South Africa: A pragmatic cluster-randomized trial (Kharitode TB). PLoS Med. 2019 Apr 30;16(4):e1002796.
16. Bajema KL, Bassett IV, Coleman SM, Ross D, Freedberg KA, Wald A, Drain PK. Subclinical tuberculosis among adults with HIV clinical features and outcomes in a South African cohort. BMC Infect Dis. 2019 Jan 5;19(1):14. doi: 10.1186/s12879-018-3614-7. PubMed PMID: 30611192; PubMed Central PMCID: PMC6321698.
17. Bassett IV, Wang B, Chetty S, Giddy J, Losina E, Mazibuko M, et al. Intensive tuberculosis screening for HIV-infected patients starting antiretroviral therapy in Durban. South Africa Clin Infect Dis. 2010;51(7):823–9.
18. Kerkhoff AD, Wood R, Lowe DM, Vogt M, Lawn SD. Blood neutrophil counts in HIV-infected patients with pulmonary tuberculosis: association with sputum mycobacterial load. PLoS One. 2013;8(7):e67956.
19. Ahmad Khan F, Verkuijl S, Parrish A, Chikwava F, Ntumy R, El-Sadr W, et al. Performance of symptom-based tuberculosis screening among people living with HIV: not as great as hoped. AIDS. 2014;28(10):1463–72.
20. Lawn SD, Brooks SV, Kranzer K, Nicol MP, Whitelaw A, Vogt M, et al. Screening for HIV-associated tuberculosis and rifampicin resistance before antiretroviral therapy using the Xpert MTB/RIF assay: a prospective study. PLoS Med. 2011;8(7):e1001067.
21. Kranzer K, Houben RM, Glynn JR, Bekker LG, Wood R, Lawn SD. Yield of HIV-associated tuberculosis during intensified case finding in resource-limited settings: a systematic review and meta-analysis. Lancet Infect Dis. 2010 Feb;10(2):93-102.
22. Human Sciences Research Council (HSRC). National Tuberculosis Prevalence Survey underway. February 2018. Available at: http://www.hsrc.ac.za/en/media-briefs/saph/national-tb-prevalance-survey-underway
23. Swindells S, Komarow L, Tripathy S, Cain KP, MacGregor RR, Achkar JM, et al. Screening for pulmonary tuberculosis in HIV-infected individuals: AIDS Clinical Trials Group protocol A5253. Int J Tuberc Lung Dis. 2013;17(4):532–9.
24. Walker NF, Stek C, Wasserman S, Wilkinson RJ, Meintjes G. The tuberculosis-associated immune reconstitution inflammatory syndrome: recent advances in clinical and pathogenesis research. Curr Opin HIV AIDS. 2018 Nov;13(6):512-521.
25. Manabe YC, Campbell JD, Sydnor E, Moore RD. Immune reconstitution inflammatory syndrome: risk factors and treatment implications. J Acquir Immune Defic Syndr. 2007 Dec 1;46(4):456-62.
26. Cain KP, McCarthy KD, Heilig CM, Monkongdee P, Tasaneeyapan T, Kanara N, et al. An algorithm for tuberculosis screening and diagnosis in people with HIV. N Engl J Med. 2010 Feb 25;362(8):707-16.
27. Huerga H et al. Diagnostic value of the urine lipoarabinomannan assay in HIV-positive, ambulatory patients with CD4 below 200 cells/µl in 2 low-resource settings: a prospective observational study. PLoS Medicine 16(4): e1002792, 2019.

For peer review only - http://bmjopen.bmj.com/site/about/guidelines.xhtml

28. Drain PK, Gounder L, Sahid F, Moosa MY. Rapid Urine LAM Testing Improves Diagnosis of Expectorated Smear-Negative Pulmonary Tuberculosis in an HIV-endemic Region. Sci Rep. 2016 Feb 11;6:19992.

For peer review only

For peer review only - http://bmjopen.bmj.com/site/about/guidelines.xhtml

Figure 1. TB testing flow chart at study enrollment among 240 SLATE I intervention arm participants Kenya

Figure 2. TB testing flow chart at study enrollment among 298 SLATE I intervention arm participants South Africa

Figure 3. TB testing flow chart at study enrollment among 296 SLATE II intervention arm participants South Africa

Figure 4. Sensitivity, specificity and positive predictive value (with 95% confidence intervals) for the WHO four symptom screen using SLATE II data.

Supplemental Fig 1. SLATE I algorithm to support same-day HIV treatment initiation [7].

Supplementary Fig 2. SLATE II algorithm to support same-day HIV treatment initiation [8].

For peer review only

For peer review only - http://bmjopen.bmj.com/site/about/guidelines.xhtml

Table 1. Self-reported TB symptoms, TB diagnosis, and TB treatment uptake among patients who screened out due to TB symptoms in the intervention arms of the SLATE I and SLATE II trials

Variable (% responding yes)	Kenya (SLATE I) (N = 240)	South Africa (SLATE I) (N = 298)	South Africa (SLATE II) (N = 296)
	n (%)	n (%)	n (%)
**Screened for TB**	240 (100)	298 (100)	296 (100)
**Currently on TB treatment***	4 (2)	2 (1)	0 (0)
**1 or more TB symptoms**	90 (38)	105 (35)	140 (47)
**Symptoms reported (among patients with 1 or more symptoms)**			
Cough (current)	75 (83)	71 (68)	76 (54)
Fever	53 (59)	45 (43)	20 (14)
Night sweats	56 (62)	44 (42)	29 (21)
Weight loss	72 (80)	76 (72)	96 (69)
**Number of symptoms reported (n)**			
1 symptom	13 (14)	36 (34)	89 (64)
2 symptoms	18 (20)	27 (26)	32 (23)
3 symptoms	29 (32)	22 (21)	8 (6)
4 symptoms	30 (33)	20 (19)	11 (8)
**TB test performed in symptomatic patients**	63 (70)	60 (57)	111 (79)
**Positive TB tests among symptomatic patients**	12 (13)	9 (9)	6 (5)
**Symptoms among those testing positive**			
Cough (current)	12 (100)	9 (100)	6 (86)
Fever	9 (75)	7 (78)	4 (57)
Night sweats	12 (100)	7 (78)	4 (57)
Weight loss	10 (83)	9 (100)	5 (71)

*Excluded from analyses

For peer review only - http://bmjopen.bmj.com/site/about/guidelines.xhtml

Figure 1. TB testing flow chart at study enrollment among 240 SLATE I intervention arm participants Kenya

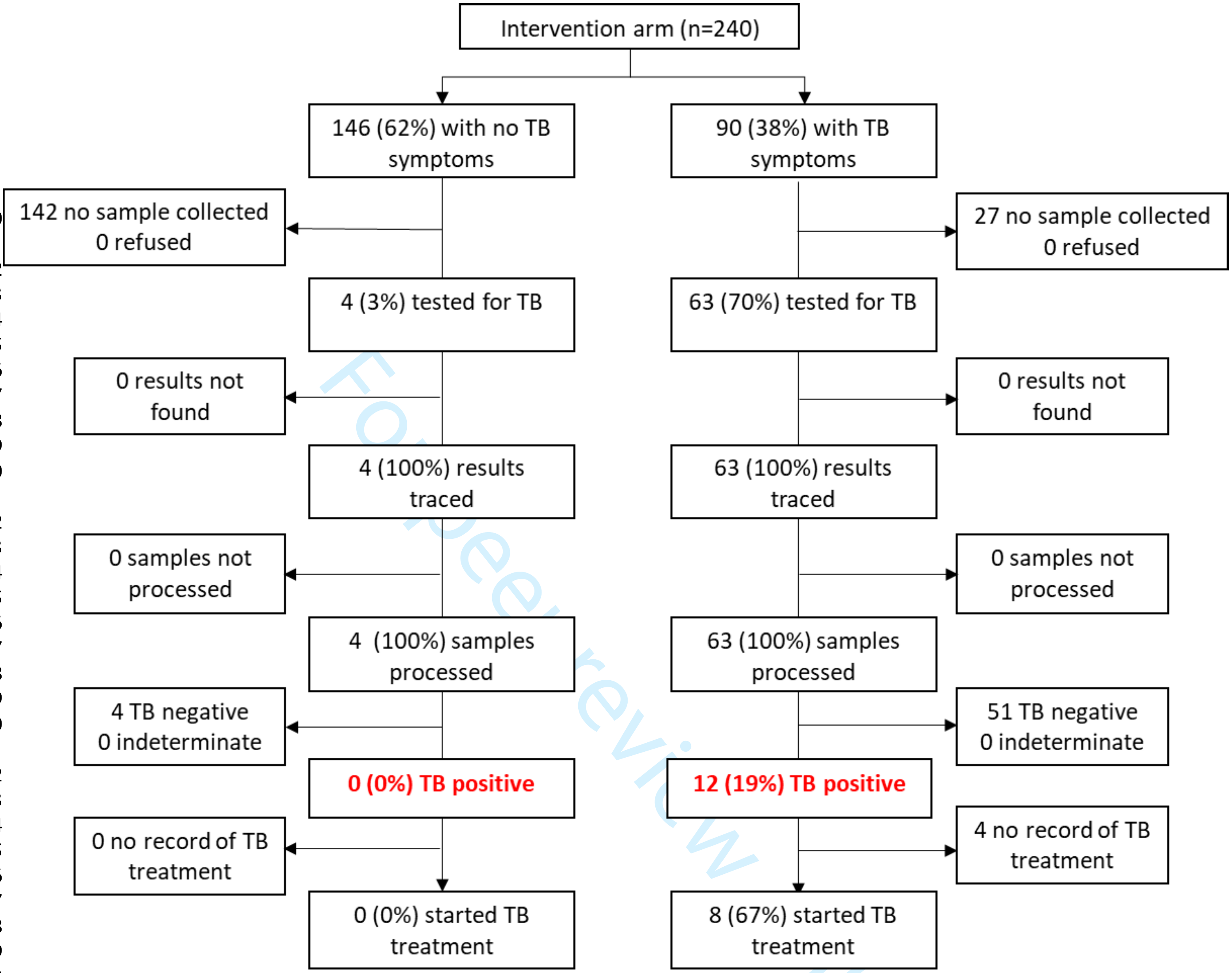

For peer review only - http://bmjopen.bmj.com/site/about/guidelines.xhtml

Figure 2. TB testing flow chart at study enrollment among 298 SLATE I intervention arm participants South Africa

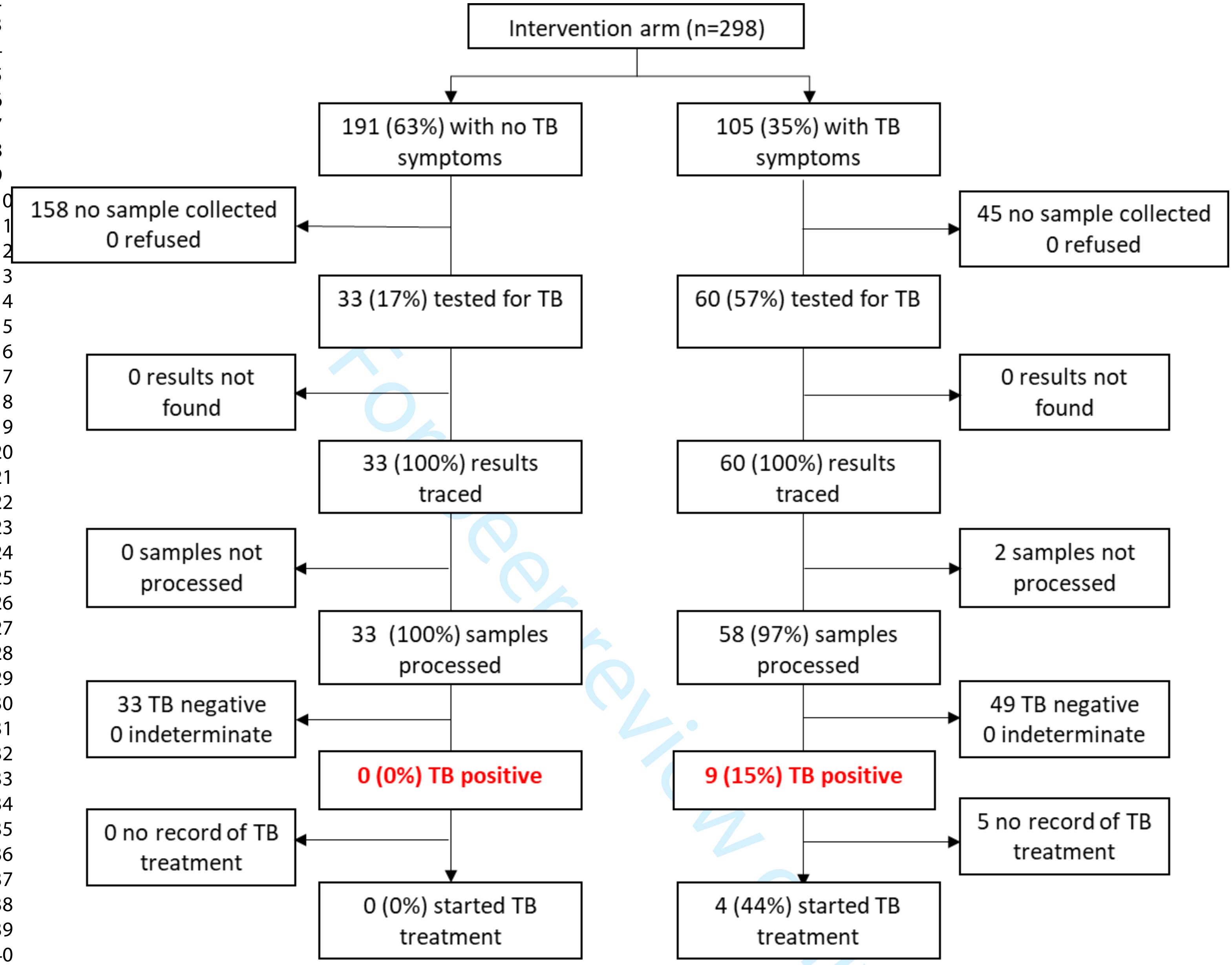

For peer review only

Figure 3. TB testing flow chart at study enrollment among 296 SLATE II intervention arm participants South Africa

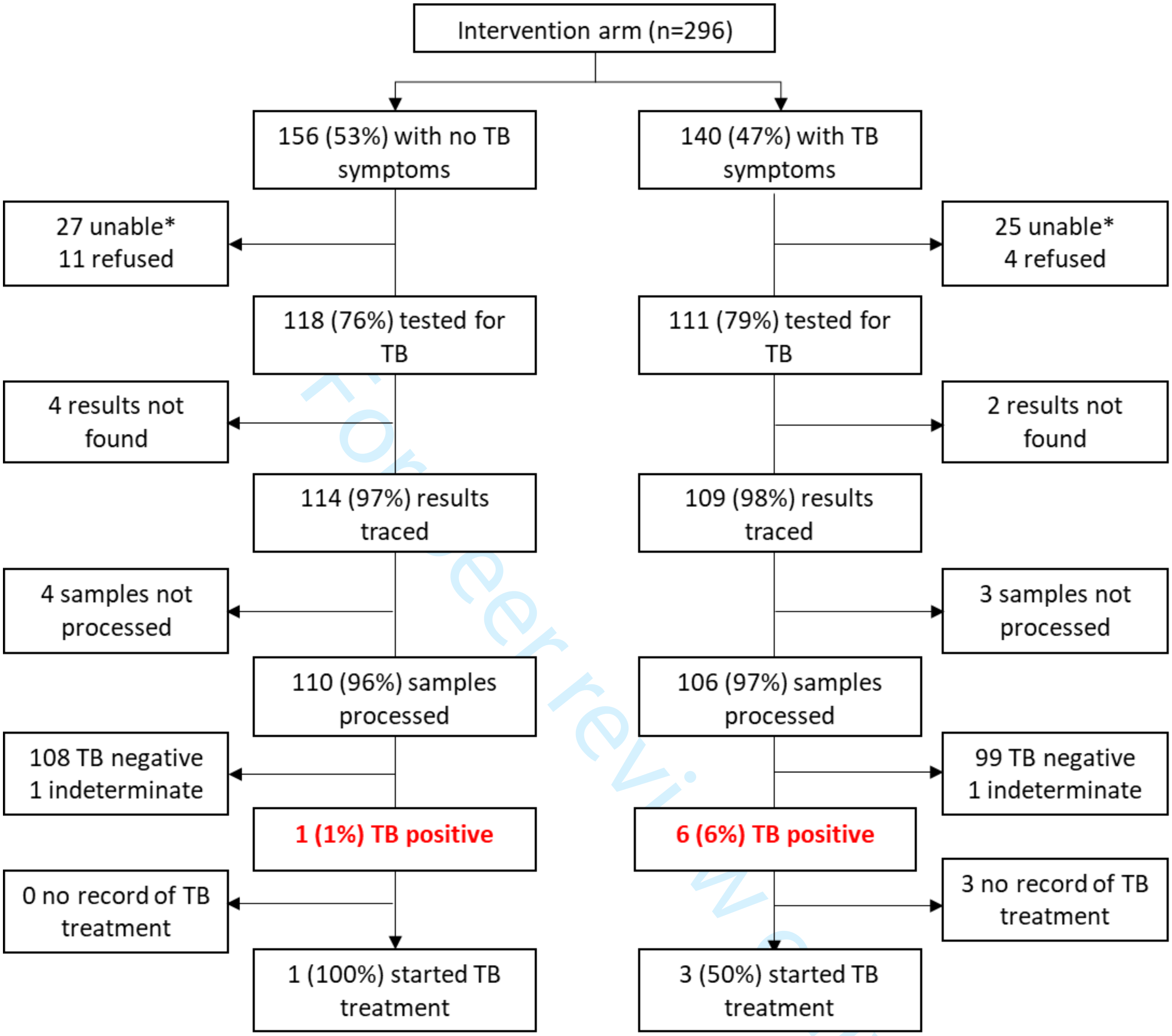

*unable to collect a sputum sample from patient

For peer review only - http://bmjopen.bmj.com/site/about/guidelines.xhtml

Figure 4. Sensitivity, specificity and positive predictive value (with 95% confidence intervals) for the WHO four symptom screen using SLATE II data.

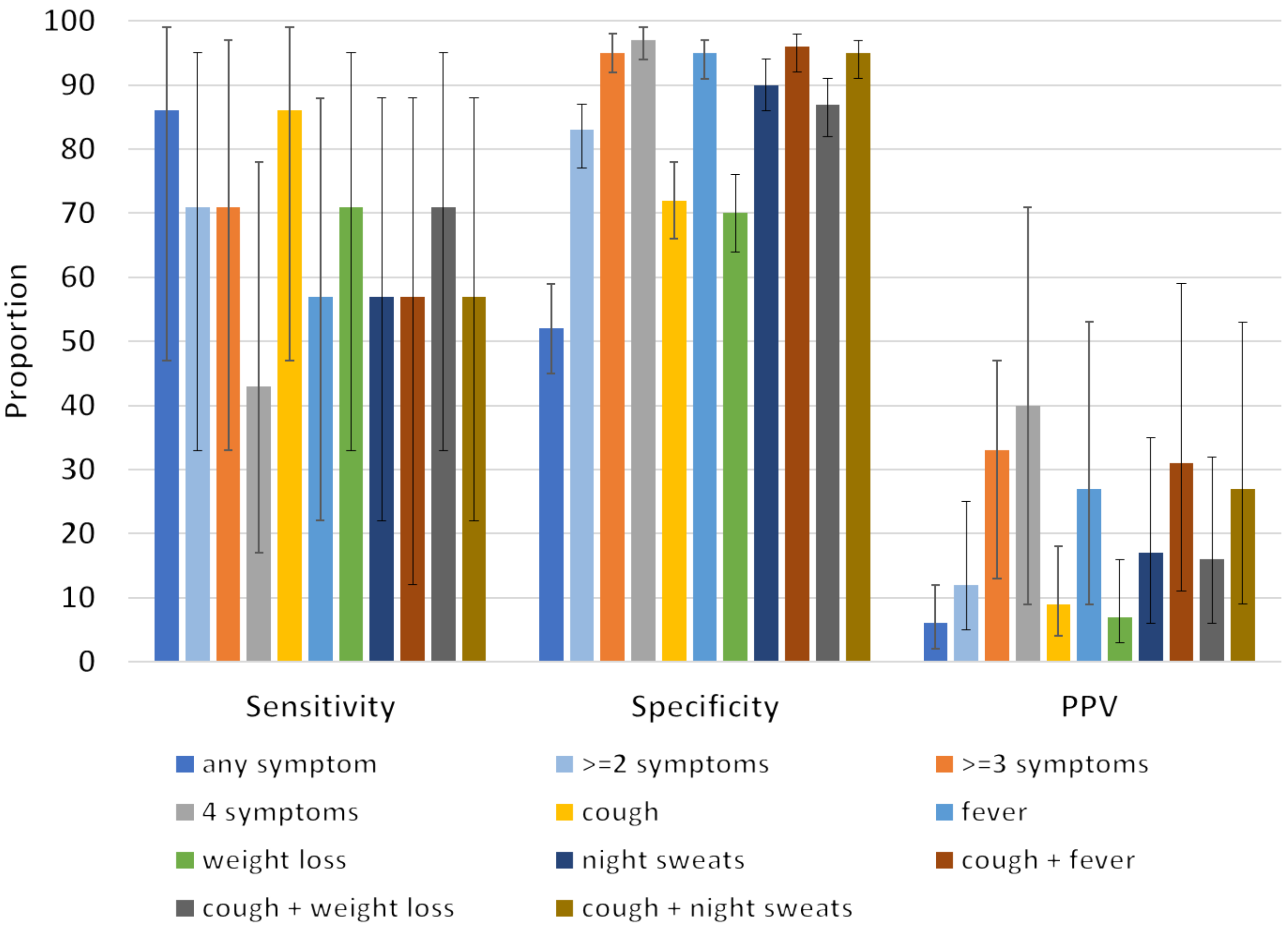

*Negative predictive values are not shown as they remain consistent at above 98% for all analyses.

For peer review only - http://bmjopen.bmj.com/site/about/guidelines.xhtml

Supplementary Fig 1. SLATE I algorithm to support same-day HIV treatment initiation [7]

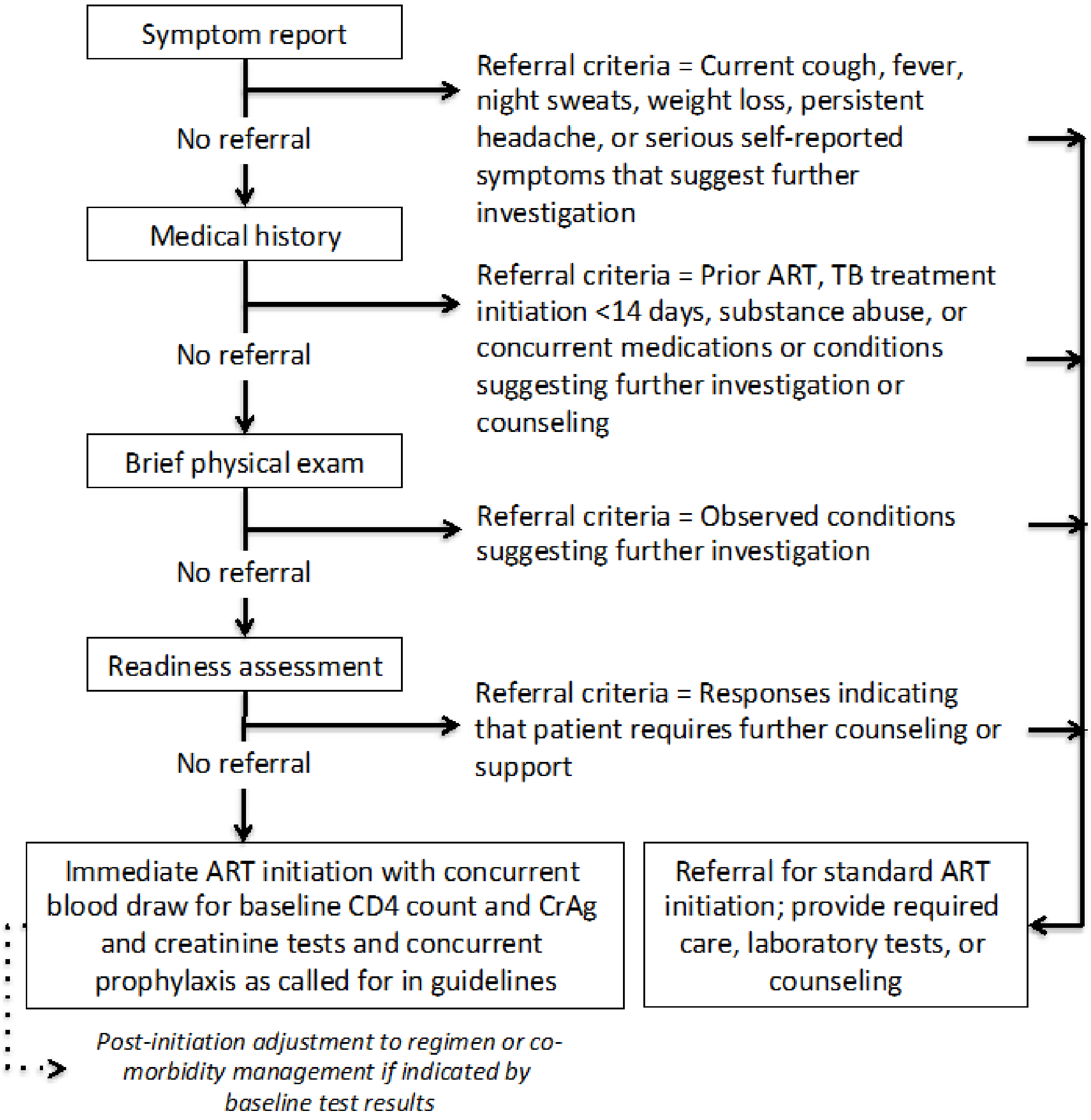

Supplementary Fig 2. SLATE II algorithm to support same-day HIV treatment initiation [8]

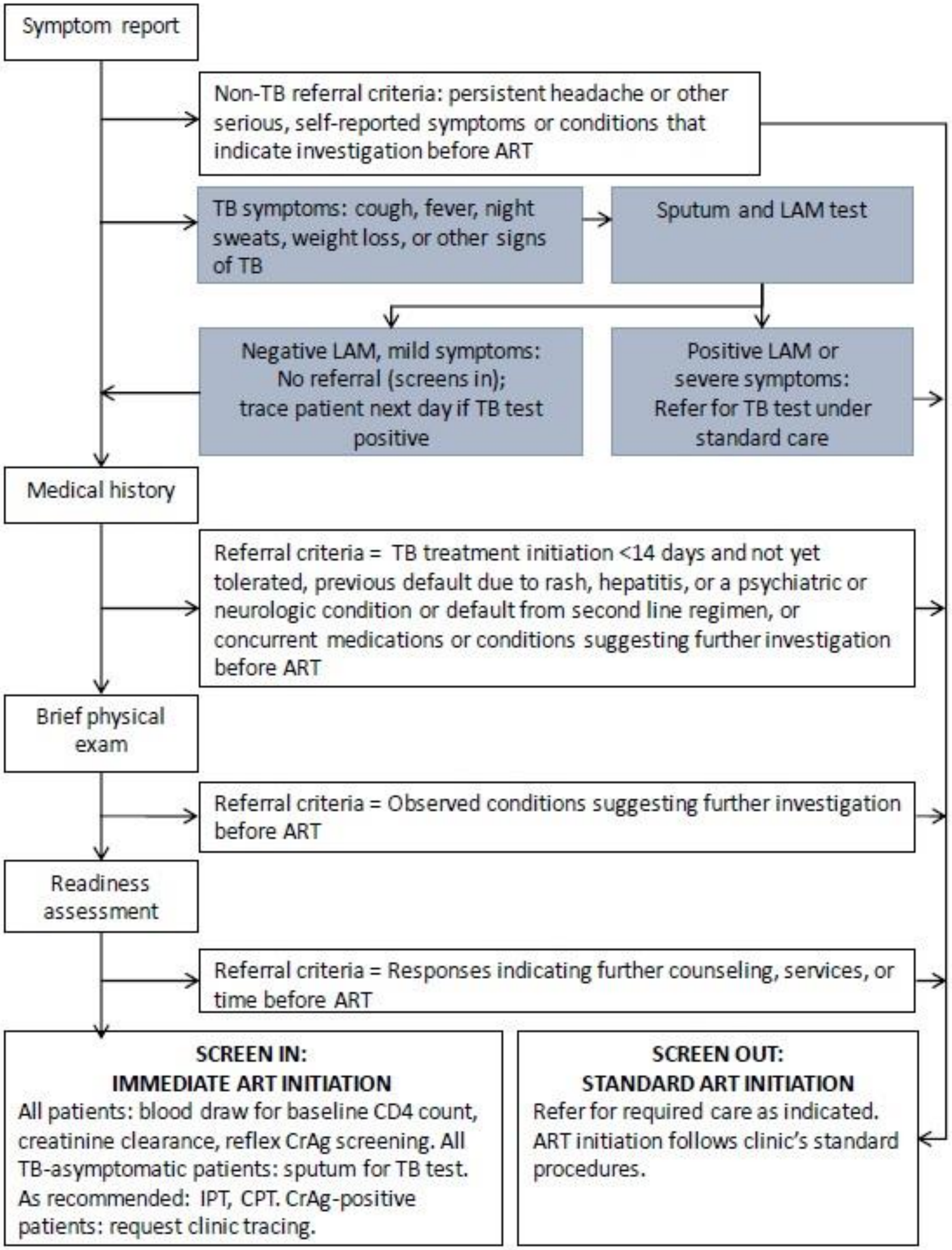

For peer review only - http://bmjopen.bmj.com/site/about/guidelines.xhtml

Supplementary Table 1. Characteristics of patients at enrollment in Kenya and South Africa SLATE I and SLATE II trials

Variables†	Categories	Intervention arm Kenya (SLATE I) (N = 240)	Intervention arm South Africa (SLATE I) (N = 298)	Intervention arm South Africa (SLATE II) (N = 296)
		n (%)	n (%)	n (%)
Period of enrollment		July 13, 2017-April 27, 2018	March 6, 2017-July 28, 2017	March 14-Sept 18, 2018
Sex	Female	142 (59)	189 (63)	189 (64)
Age	Median (IQR)	36 (29, 44)	34 (29, 41)	35 (29, 41)
CD4 count at enrollment (cells/mm^3)	Median (IQR)	272 (124, 522)	275 (132, 459)	294 (135, 464)
Location of current residence	Town (urban)	35 (15)	18 (6)	31 (10)
	Peri-urban	66 (28)	299 (89)	265 (90)
	Rural home or village	139 (58	14 (5)	0 (0)
Current house is primary residence	Yes	154 (64)	141 (47)	128 (43)
Number other persons in house	Median (IQR)	3 (2, 5)	2 (1, 3)	1 (1, 3)
Usual activity when well	Formal employment	18 (8)	82 (28)	69 (23)
	Informal sector work	161 (67)	80 (27)	70 (24)
	Unemployed, looking for work	12 (5)	107 (36)	138 (47)
	Other	9 (4)	29 (10)	12 (4)
Transport mode to clinic today (multiple choices possible)	Minibus or taxi	118 (49)	122 (41)	110 (37)
	Walking	46 (19)	156 (53)	161 (54)
	Private car	11 (5)	19 (19)	25 (8)
	Motorbike	137 (57)	0 (0)	0 (0)

For peer review only

For peer review only - http://bmjopen.bmj.com/site/about/guidelines.xhtml

Supplementary Table 2. Median CD4 count (cells/mm^3) for patients with and without TB symptoms and those with TB diagnosis in SLATE I and SLATE II trials

Variable	Median CD4 Count Intervention arm Kenya (SLATE I)		Median CD4 Count Intervention arm South Africa (SLATE I)		Median CD4 Count Intervention arm South Africa (SLATE II)	
	n (%)	Median (IQR)	n (%)	Median (IQR)	n (%)	Median (IQR)
**No TB symptoms**	148 (62)	357 (191-632)	191 (63)	307 (141-486)	156 (53)	405 (210-547)
**TB symptom positive**	90 (38)	152 (64-329)	105 (37)	245 (125-425)	140 (47)	175 (89-365)
**TB symptom presence**						
Cough (current)	75 (83)	144 (58-319)	71 (68)	274 (132-433)	76 (54)	157 (68-416)
Fever	53 (59)	136 (49-316)	45 (43)	230 (78-368)	20 (14)	110 (73-203)
Night sweats	56 (62)	215 (56-388)	44 (42)	229 (176-368)	29 (21)	135 (101-367)
Weight loss	72 (80)	146 (53-319)	76 (72)	251 (133-433)	96 (69)	157 (68-337)
**TB symptoms**						
1 symptom	13 (14)	207 (147-317)	36 (34)	188 (72-295)	89 (64)	250 (98-369)
2 symptoms	18 (20)	95 (64-232)	27 (26)	373 (227-520)	32 (23)	137 (76-342)
3 symptoms	29 (32)	232 (72-406)	22 (21)	216 (76-368)	8 (6)	165 (63-350)
4 symptoms	30 (33)	116 (24-277)	20 (19)	267 (180-423)	11 (8)	106 (68-157)
**Symptom prevalence in those diagnosed with TB**	**n=12**		**n=9**		**n=7**	
Cough (current)	12 (100)	124 (12-150)	9 (100)	251 (47-288)	6 (86)	92 (60-107)
Fever	9 (75)	114 (12-150)	7 (78)	201 (18-339)	4 (57)	92 (69-107)
Night sweats	12 (100)	142 (55-224)	7 (78)	201 (18-288)	4 (57)	69 (40-92)
Weight loss	10 (83)	124 (12-150)	9 (100)	251 (47-288)	5 (71)	78 (60-106)

For peer review only - http://bmjopen.bmj.com/site/about/guidelines.xhtml
